# Protective alleles and precision healthcare in crewed spaceflight

**DOI:** 10.1038/s41467-024-49423-6

**Published:** 2024-07-22

**Authors:** Lindsay A. Rutter, Matthew J. MacKay, Henry Cope, Nathaniel J. Szewczyk, JangKeun Kim, Eliah Overbey, Braden T. Tierney, Masafumi Muratani, Ben Lamm, Daniela Bezdan, Amber M. Paul, Michael A. Schmidt, George M. Church, Stefania Giacomello, Christopher E. Mason

**Affiliations:** 1 Transborder Medical Research Center, University of Tsukuba, Ibaraki, 305-8575 Japan; 2 Department of Genome Biology, Institute of Medicine, University of Tsukuba, Ibaraki, 305-8575 Japan; 3 Department of Physiology and Biophysics, Weill Cornell Medicine, New York, NY 10065 USA; 4 The HRH Prince Alwaleed Bin Talal Bin Abdulaziz Alsaud Institute for Computational Biomedicine, Weill Cornell Medicine, New York, NY 10021 USA; 5 The WorldQuant Initiative for Quantitative Prediction, Weill Cornell Medicine, New York, NY 10065 USA; 6 School of Medicine, University of Nottingham, Nottingham, DE22 3DT UK; 7 Ohio Musculoskeletal and Neurological Institute (OMNI), Heritage College of Osteopathic Medicine, Ohio University, Athens, OH 45701 USA; 8 Colossal Biosciences, 1401 Lavaca St, Unit #155 Austin, Austin, TX 78701 USA; 9 Institute of Medical Genetics and Applied Genomics, University of Tübingen, Tübingen, Germany; 10 NGS Competence Center Tübingen (NCCT), University of Tübingen, Tübingen, Germany; 11 Yuri GmbH, Meckenbeuren, Germany; 12 Embry-Riddle Aeronautical University, Department of Human Factors and Behavioral Neurobiology, Daytona Beach, FL 32114 USA; 13 Sovaris Aerospace, Boulder, CO 80302 USA; 14 Advanced Pattern Analysis & Human Performance Group, Boulder, CO 80302 USA; 15 GC Therapeutics Inc, Cambridge, MA 02139 USA; 16 Department of Genetics, Harvard Medical School, Boston, MA 02115 USA; 17 Wyss Institute for Biologically Inspired Engineering, Harvard University, Cambridge, MA 02115 USA; 18 SciLifeLab, KTH Royal Institute of Technology, Stockholm, 17165 Sweden; 19 The Feil Family Brain and Mind Research Institute, Weill Cornell Medicine, New York, NY 10065 USA; 20 Present Address: School of Chemistry, University of Glasgow, Glasgow, G12 8QQ UK

**Keywords:** Rare variants, Personalized medicine

## Abstract

Common and rare alleles are now being annotated across millions of human genomes, and omics technologies are increasingly being used to develop health and treatment recommendations. However, these alleles have not yet been systematically characterized relative to aerospace medicine. Here, we review published alleles naturally found in human cohorts that have a likely protective effect, which is linked to decreased cancer risk and improved bone, muscular, and cardiovascular health. Although some technical and ethical challenges remain, research into these protective mechanisms could translate into improved nutrition, exercise, and health recommendations for crew members during deep space missions.

## Abstract (web summary)

As space travel promises to become a reality for more humans, insights from human genetics could serve to inform space medicine. Here, the authors overview genetic variants that might confer a protective effect in space, and ethical and technical challenges to translating these findings.

## Introduction

**Fig. 1:**
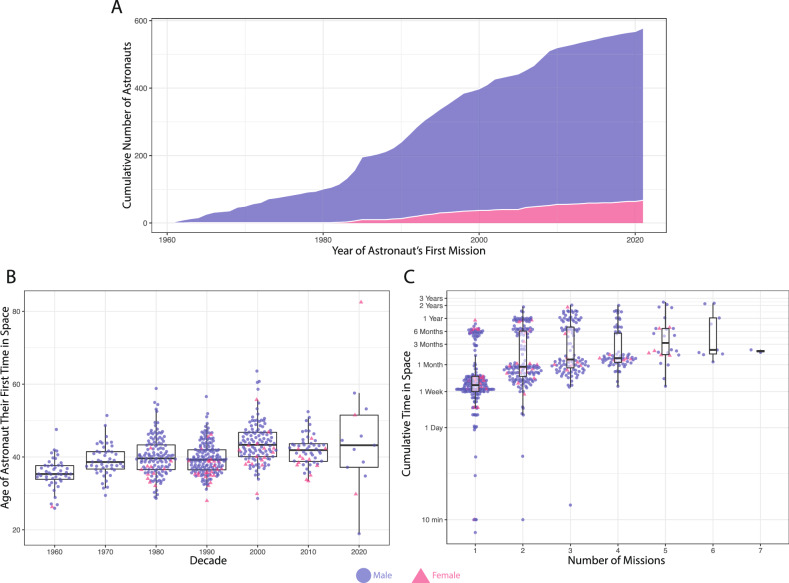
Increasing time and breadth of astronauts. **A** The cumulative number of astronauts who have been in space, plotted by the year of their first mission. **B** Boxplots representing the age of astronauts on their first mission plotted by the decade of their first mission. **C** Boxplots representing the cumulative amount of time an astronaut spent in space plotted against the number of missions in which they have participated. Blue signifies male and pink signifies female astronauts in all plots. Data was scraped from supercluster.com on September 20th, 2021. Only astronauts who spent time in space and crossed the Kármán line are displayed.

Humankind has entered a new chapter of deep space exploration, with numerous institutions worldwide publicly aspiring toward a sustainable human presence on the Moon and Mars, which is also broadening the diversity of humans in space. In the past two years, the first person with a prosthetic leg entered space (Inspiration4), and the first person with a physical disability was selected for professional astronaut training by the European Space Agency (ESA). The next few years of spaceflight also feature planned missions to the circumlunar environment after a half-century hiatus, including the first crewed commercial lunar flyby (a team of artists and civilians for the dearMoon project), further broadening the range of people going into space. Moreover, while a steady increase in female space explorers began in 1982, the first mission to land the first woman and person of color on the Moon will finally occur in the Artemis III mission (Fig. 1A), coincident with a broadening of the age ranges (Fig. 1B) and mission durations (Fig. 1C) of the astronauts.

Of note, only 24 humans have voyaged into the more hazardous conditions outside low-Earth orbit (LEO), all during the Apollo program. The longest Apollo mission lasted only 12 days, and all adventurers were white men between ages 35 and 47, all of whom had passed rigorous physical fitness tests. Consequently, the objective of sending humankind to unexplored horizons over unmatched timescales evokes critical questions about how the human body will respond to new extremes, as well as how to best protect the health of the broader space-faring population. In the coming decades, public and professional space explorers will likely embark on multi-year missions to the Moon and Mars, and this can lead to a more equitable vision of crewed space exploration. Specifically, the space community can democratize vital aspects of spaceflight, ranging from tailored space suit designs, precision health recommendations, and customized countermeasures^1,2^. Risks for these crews include radiation-induced cancers, space-induced osteopenia, challenges with pregnancy and child development, and changes for human bodies in Martian- and Lunar-based communities.

The same motivations that drive precision healthcare on Earth could be useful for these new populations in space, where molecular data could be employed to guide therapy and mission recommendations. Multipronged countermeasures that are both environmental (such as advanced spacesuits, spacecrafts, and space habitats) and biomedical (such as omics-based nutrition, exercise, and lifestyle recommendations) likely represent the best contenders to optimize health across a more inclusive gamut of humanity in space. In this paper, we highlight omics technologies as one potential component toward improving prospective health countermeasures in space^2^.

Omics modeling tools assess multiple biological responses (including genomics, epigenomics, transcriptomics, epitranscriptomics, metagenomics, metatranscriptomics, metabolomics, and proteomics) that can converge into useful solutions for mitigating spaceflight-induced physiological deconditioning. Indeed, omics-based recommendations can be integrated alongside other countermeasures to sustain human health in space, and near real-time health monitoring in space could also one day incorporate omics^3^. We review an example set of published alleles that appear to be linked to health-protective effects, with relevance both on Earth and in space. Basic research into the protective mechanisms of such alleles may translate into improved therapeutic compound discovery and better-informed nutrition, exercise, and lifestyle recommendations, not only for space explorers, but also for those who work in challenging conditions on Earth. We conclude by discussing some of the significant scientific and ethical challenges related to these themes.

## Results

**Fig. 2:**
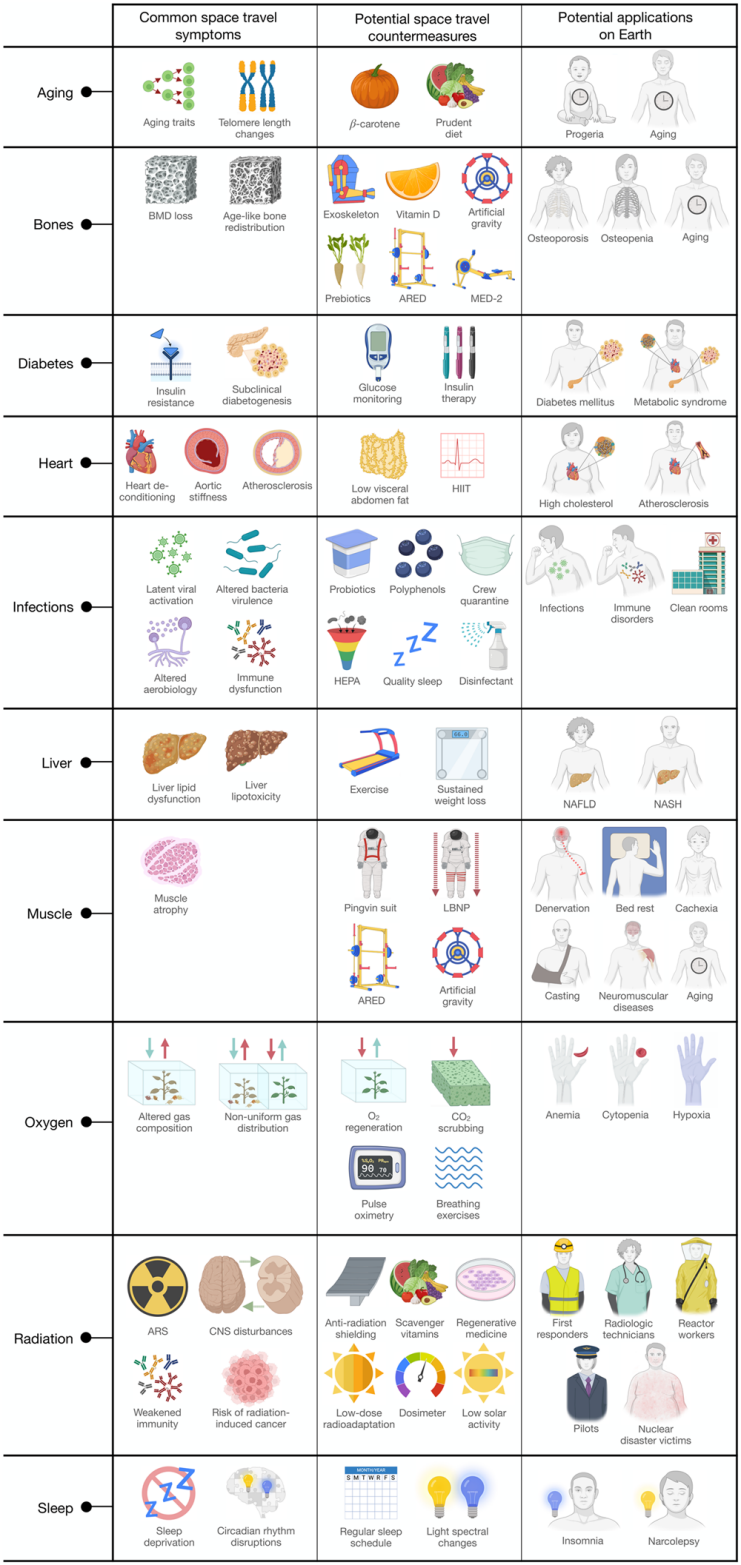
Spaceflight health risk countermeasures. Examples of health-risk categories that warrant precision countermeasures to improve the physical fitness of both spaceflight participants and terrestrial humankind. From left to right: examples of common spaceflight ailments related to the health-risk category; examples of spaceflight countermeasures related to the health-risk category; examples of terrestrial populations that could benefit from improved precision countermeasures related to the health-risk category. Created with BioRender.com released under a Creative Commons Attribution-NonCommercial-NoDerivs 4.0 International license.

Growing evidence indicates that spaceflight can elicit a range of stressful and adaptive physiological responses on the human body. Environmental harms like radiation, microgravity, and altered gas composition can jeopardize the health of the musculoskeletal, cardiovascular, digestive, nervous, integumentary, and immune systems; increase the risk of cancer, diabetes, and aging traits; and disrupt vision, sleep cycles, and cognitive behavior^1^. There are a range of known alleles across various health-risk categories (Fig. 2, Supplementary Data 1) that may warrant effective and precision countermeasures to improve the physical fitness of both spaceflight participants and terrestrial humans.

The human health challenges of crewed LEO, cis-lunar, and interplanetary missions will benefit from the development of countermeasures for optimal crew health and safety, especially for known genes that could confer a strong impact on spaceflight-related risks. Examples of known genes relevant for spaceflight include those related to altered radiation tolerance (*TP53*), hematopoiesis (*EPOR*), bone density (*LRP5*), and immune function (HLA alleles and cytokines)^4^, although there may be upper limits for adaptation^5^. As such, even when the mechanism of a phenotype is known, natural evolution with extant alleles may have a limited range for adaptation. Nonetheless, an important role of omics (and other omics modeling, such as organoids)^6,7^ is to understand risks and mechanisms at a deeper level, so that traditional protective measures (such as spacesuits, exercise equipment, and radiation shielding) can be designed in more informed ways. As a result, comprehensive aerospace healthcare programs in the future would likely integrate both omics information and traditional protective measures. We briefly illustrate this below by focusing on key examples of radiation-induced cancer and space-induced bone demineralization.

### Example 1: Space-induced radiation syndrome and cancer threat

Radiation exposure is one of the predominant health hazards of deep space missions, for both in-flight and long-term, post-flight risks. In-flight concerns include acute radiation sickness (ARS), which may include hair loss, cataracts, metabolic deficiency, and gastrointestinal dysfunction^8,9^; central nervous system (CNS) disturbances that may affect cognitive abilities, motor functions, and behavioral performance^10^; and degeneration of radiation-sensitive immunological tissues that could weaken the immune system^11^. One prominent long-term issue is the risk of fatal cancer from radiation exposure^12,13^. For context, over the course of one year, the typical human is exposed to approximately 3 milliSieverts (mSv) on Earth versus 144 mSv on the International Space Station (ISS)^14,15^, in addition to varied doses and types of ionizing radiation (IR).

Astronauts on the ISS, which is located in LEO, are exposed to IR but are mostly protected by Earth’s magnetosphere. In contrast, future deep space missions outside of LEO will expose astronauts to increased doses of radiation, with a higher flux of galactic cosmic rays (GCRs)^12^. For example, a single 500-day mission to Mars would likely expose astronauts to 1000 mSv, the career limit set by international space agencies, which corresponds to a 3% increased risk of lethality from exposure-induced cancer^16^. Recent studies that incorporated non-targeted radiation effects estimate that cancer risks from Mars missions may be higher than previously estimated^17^. It is worth noting that other celestial bodies that have been nominated for far-future human exploration may be even more dangerous: One example is Europa, which receives more than 5000 mSv of radiation each day, enough to prove deadly to humans^18^.

Current radiation countermeasures include nutrition, drugs, and anti-radiation spacecraft shielding, but these offer only partial protection^19,20^. Moreover, there are currently no FDA-approved radioprotectants that can be deployed for crews, although cytokine supplementation therapy (Neupogen, Neulasta, and Leukine) has been approved by the FDA for the treatment of the acute hematopoietic syndrome. Other possible radiation countermeasures could include radiation absorbers and nutrition (scavenger vitamins, trace elements, and minerals) (Fig. 2)^12,21^. In addition, individual susceptibility to radiation-induced cancer may be a key factor^22,23^, with individual differences observed even in early studies of radiation victims^24^, and radiosensitivity appears to be influenced by a variety of factors. These factors include known susceptibility genes (such as BReast CAncer gene (BRCA) 1 and 2 mutations), age, sex, nutrition, toxin exposure, comorbidities, inflammatory state, and viral infections^25^. At this time, the National Aeronautics and Space Administration (NASA) predicts individual risk for carcinogenesis based on statistics at the population level, and genomics and other omics data could better inform these estimates^26^. Such omics-based information for spaceflight could also be relevant for soldiers, emergency first responders, coal miners, radiologic technicians, reactor workers, pilots, and others who enter radioactive environments (Fig. 2)^27^.

### Example 2: Space-induced bone demineralization

Observations within the limited setting of LEO suggest an average bone mineral density (BMD) loss of 1–1.5% each month for weight-bearing bones^28,29^. Six-month missions on the ISS reportedly induce bone loss equivalent to two decades of aging, some of which may be long-lasting^30^; this finding has implications about missions that will last more than one year on the ISS, the Moon, and Mars. Multiyear missions, especially ones that journey outside of LEO, are harder to model, due to the absence of any comparable data. However, recent models estimate astronauts may suffer 32.4–36.8% BMD loss during missions to Mars, with up to 100% developing osteopenia, 33% developing osteoporosis, and 79% reaching fracture risk levels that NASA deems impermissible^31^. These models were developed using astronaut data inside LEO^31^, which is a critical limitation; deep space radiation is hypothesized to exacerbate the BMD loss already caused by microgravity unloading^32^. For example, cancer patients routinely experience increased bone fracture risk in areas irradiated during treatment^33^. Hence, the severity of bone damage during interplanetary missions may be even higher, once taking into account the factor of deep space radiation and calcium loss. Bone healing could also be impaired due to microgravity, where sepsis and thromboembolic blood clots could ultimately present^31^. Moreover, even though BMD loss has been shown to partially recover one year after spaceflight, recovered bone appeared to be deposited into bone architecture similar to that of older people^34^, indicating the recovery is incomplete.

With several upcoming human missions into deep space (e.g. Artemis II, III, dearMoon, and Mars missions), we will better understand the nature of space-induced bone demineralization. Those data could likely improve countermeasures and the incorporation of exercise, pharmacology, and nutrition protocols (Fig. 2). For example, the Advanced Resistive Exercise Device (ARED) improves the health of astronauts by reducing the magnitude of common space health ailments, including BMD decreases^35^, isokinetic strength decreases^36^, and immune system dysregulation^37^. The ARED may confer more pronounced physiological benefits than its predecessor, the interim Resistive Exercise Device (iRED), which only offered resistance exercise at lower quality and quantity loads. Unfortunately, current exercise spaceflight devices may not practically be deployed in upcoming deep space vessels due to limited cargo volume. As such, flight exercise hardware may need to be scaled down for compatibility, and the feasibility of next-generation miniaturized equipment (such as the Miniature Exercise Device-2 (MED-2)) is currently being assessed^38^.

Vitamin D is another key mediator of bone health through calcium absorption and bone mineralization, and represents a relevant target for upcoming missions. Prior to 2006, crews supplemented with ~400 International Units (IU) of vitamin D each day during 3–6 months missions still showed decreases in vitamin D status postflight^39^. When ground-based studies in Antarctica and Johnson Space Center verified that daily vitamin D doses of 800–2000 IU were safe and could maintain vitamin D status for 3–6 months in settings devoid of ultraviolet (UV) light exposure, vitamin D supplementation recommendations for space crew in similar missions were increased in 2006 from daily doses of 400 to 800 IU^40^. Despite such evidence-based improvements in dietary and exercise countermeasures, BMD loss remains an unsolved health hazard in spaceflight that will likely be more problematic for future long-term missions. Here again, omics data analysis could potentially further discover and improve countermeasures. While current equations that predict BMD loss consider population-level differences with factors of ethnicity, sex, and age^31^, research suggests that genetics may account for 60–80% of bone mass variability, and protective genes like *LRP5* can help guide risk for astronauts. Naturally, any valuable omics-based countermeasures developed for bone health in space could spur innovations that benefit populations on Earth who suffer from bone loss, including the elderly, postmenopausal people, people with osteoporosis, and cancer patients who endure incidental bone loss after radiation treatment (Fig. 2).

### Studying omics for improved spaceflight safety

**Fig. 3:**
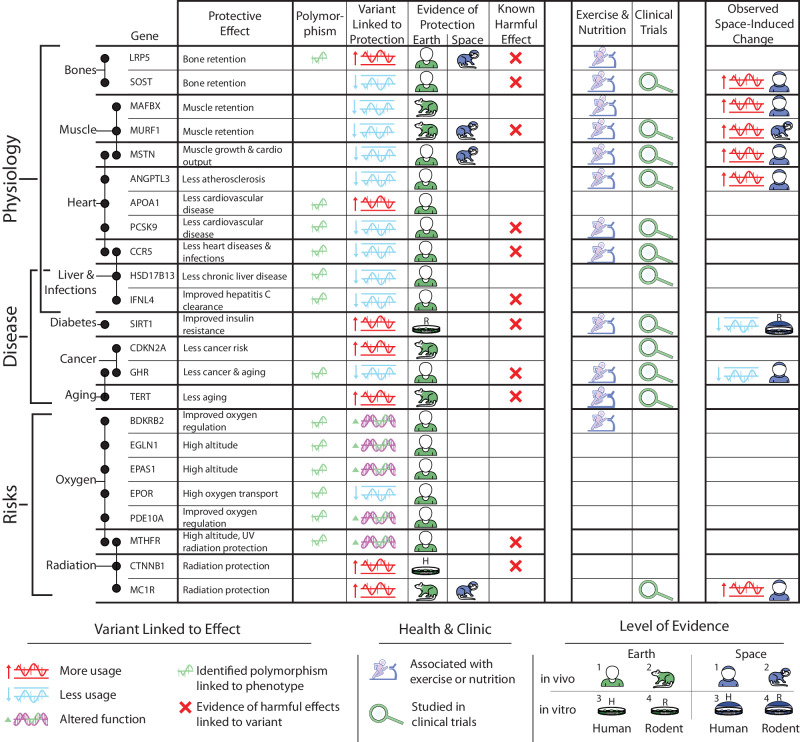
Literature review of evidence for protective alleles. Literature review and its key findings for a subset of alleles that may be linked to health protective effects, many of which occur naturally in the human population. From left to right: the gene name; example protective phenotypes; whether a polymorphism has been linked to the protective phenotype; whether the protective phenotype typically relates to over, under, or variant function of the gene; the level of evidence of protection for the variant on Earth (green icons) or in space (blue icons); and examples of known harmful associations with this allele. We further include if the gene or its related pathway can be targeted with exercise or nutrition (purple icon), has been studied in clinical trials (green icon), or has been observed to alter function during spaceflight. Only the highest level of evidence we found is displayed, with in vivo being defined as stronger evidence than in vitro, and health benefits for human subjects being defined as higher evidence than health benefits for rodent subjects. Empty cells indicate instances in which we were unable to find moderate evidence based on current literature. Partly created with BioRender.com released under a Creative Commons Attribution-NonCommercial-NoDerivs 4.0 International license. More details and literature references are provided in the Supplementary Data.

Of note, there are some published alleles (Fig. 3) that occur naturally in human populations, and which have been linked to protective mechanisms with possible relevance to spaceflight healthcare, which could help guide the countermeasure examples described above. The protective effects of some of these alleles appear to mechanistically overlap with nutrition and exercise interventions, and have motivated new clinical trials (Fig. 3). Hence, basic scientific research into the protective mechanisms of these alleles could one day translate into improved therapeutic compound discovery and better-informed nutrition, exercise, and lifestyle recommendations for crew members to maintain their health during deep space exploration. After reviewing these published alleles and their promising implications, we then follow with examples of the numerous technical and ethical difficulties involved with basic scientific research into the protective mechanisms linked to these alleles.

### Human cohorts: Individuals

Researchers discovered several of the alleles (Fig. 3) with protective mechanisms by studying special human cohorts, especially individuals with rare phenotypes. For example, a German toddler appeared “extraordinarily muscular” and was eventually diagnosed with a mutation in the myostatin-producing *MSTN* (*GDF8*) gene^41^. The loss-of-function mutation translated into an absence of mature myostatin for the patient, who experienced gross muscle hypertrophy, with quadricep muscles 7.2 standard deviations (SD) above average and subcutaneous fat thickness 2.88 SD below the expected mean^41^. *MSTN* targets are also being utilized in phase 2 clinical trials of humanized monoclonal *MSTN* antibodies, which confer increased muscle mass and strength in older individuals with recent falls^42^, and thus could represent applications for astronauts as well. Resistance training and creatine supplementation to decrease myostatin serum levels have also been deployed based on individual profiles^43^, which represent a non-omics intervention option for spaceflight.

### Human cohorts: Families

Several other alleles have come to light through studying families with protective attributes. For example, genes linked to stronger bones (*LRP5*) and higher oxygen transport (*EPOR*) have been found in several family-based studies, and these protective pathways may be promising for spaceflight healthcare (Fig. 3). Specifically, the *LRP5* G171V mutation was discovered when family members showed strikingly high BMD in clinical screenings while reporting difficulty staying afloat while swimming and no history of bone fractures^44,45^. The gain-of-function variant induces BMD about five times greater than the mean of the general population.

The *EPOR* W439X mutation was found through linkage analysis of almost 100 family members^46^. The mutation elicits the beneficial symptoms of erythrocytosis (such as increased hemoglobin count, hematocrit count, and oxygen transport capacity) with only mild or minimal negative effects^46^. Interestingly, one proband, who had hemoglobin >200 g/L since childhood, was an Olympic gold medalist in cross-country skiing^46^. Annotating the network underpinnings of this allele could unravel new methods to combat clinical conditions that space travelers routinely suffer when exposed to altered gas composition in spacecraft.

### Human cohorts: Isolated populations

Isolated populations have also revealed alleles linked to alleviated clinical manifestations of diseases like atherosclerosis and cancer (Fig. 3). For instance, in a patient from Limone sul Garda (an isolated village in Northern Italy), the *APOA1* R173C mutation was associated with low high-density lipoprotein (HDL) levels and elevated triglycerides, but unexpectedly no atherosclerotic disease^47^. When the complete Limone sul Garda population was sampled (*n* = 1000 total), 33 living carriers of the “ApoA-1 Milano” mutation were identified^47^, all with low HDL, and yet low incidence of heart disease. Genealogical analysis using church records suggested they were all possible descendants of one man born in the village in 1780^48^. Patients with acute coronary syndromes intravenously administered R173C phospholipid complexes show significant regression of coronary atherosclerosis^49^.

Studying the protective effects linked to *APOA1* could reveal new methods to mitigate the risk of myocardial infarction and atherosclerosis, especially since astronauts endure above-average radiation exposure^50,51^. However, it is still unclear whether spaceflight, particularly long-duration missions outside LEO, may increase long-term atherosclerotic risk^52^. Statins may reduce radiation-induced atherosclerosis, but published results are thus not conclusive. The absence of suitable prophylaxes for atherosclerosis risk in both terrestrial humans exposed to radiation and astronauts exposed to space radiation motivates the thorough analysis of the *APOA1* R173C mechanism to potentially uncover safe and effective therapeutic compounds.

Additional examples of alleles linked to protective effects found in families include those related to Laron syndrome and genetic markers in Longevity Blue Zone (LBZ) populations. Laron syndrome is a rare condition that has been disproportionately reported in remote and inbred villages in the Loja province of Ecuador^53^. Patients typically have mutations in the *GHR* gene that are linked to growth hormone deficiency and decreased stature but are also linked to extended health span by potentially mitigating cancer and diabetes^54^. Given that cancer is a primary cause of death and a major health risk in upcoming long-term spaceflight, understanding the physiological underpinnings of *GHR* mutations associated with Laron syndrome could lead to potential anti-cancer therapies.

LBZs are areas where the local population consists of unusually high numbers of elderly individuals. These LBZ populations have been identified in Sardinia and the Greek island of Ikaria. When compared to reference populations in Italy and Greece, LBZ populations show lower frequencies in genetic risk markers in *APOE4*, *APOE2*, and the TT allele of *FOXO3*, which are associated with an increased risk for Alzheimer’s disease and decreased cognitive function^55^.

### Human cohorts: Populations adapted to extreme environments

Research on human adaptation to extreme environments has also revealed alleles with health implications applicable to spaceflight (Fig. 3), including deep-diving adaptations in indigenous Bajau people and high-altitude adaptation in indigenous people of the Tibetan Plateau. Both populations have adapted to lifestyles in which the gas composition of their surroundings is uniquely altered compared to most human populations, potentially informing new approaches to combat physiological maladies that many astronauts endure due to altered gas composition (e.g. hypercapnia) on the ISS.

The Bajau people, sometimes referred to as “Sea Nomads”, have lived marine hunter-gatherer lifestyles in Southeast Asian seas for over 1000 years^56^. They are renowned for their remarkable breath-holding abilities, diving up to 73 meters using only wooden goggles and spending up to five hours each day apnea diving (breath-hold diving). One recent study found that Bajau people have significantly larger spleens than Saluan people, geographical neighbors with minimal marine-related lifestyles^57^. The likely cause is natural selection on allelic variants in the *PDE10A* gene, which are associated with larger spleen size, providing them with additional reserves of oxygenated red blood cells. The study also found evidence of strong selection for variants in the *BDKRB2* gene, thought to increase peripheral vasoconstriction to preferentially redirect blood flow to critical tissues like the brain, heart, and lungs. Other candidate genes that appeared to be selected in the Bajau population included *FAM178B*, which encodes a protein that helps prevent the buildup of carbon dioxide, and *CACNA1A*, which regulates response to hypoxic conditions^58^.

Insights into the genotypic physiology of hypoxia have not only emerged from populations who adapted to the seas, but also from those who adapted to the mountains. For millennia, people have lived on the Tibetan Plateau, which is surrounded by the two highest summits on Earth (Mount Everest and K2). The plateau averages ~4,000 meters above sea level and has 60% of the oxygen concentration and 130% of the UV radiation compared to sea level^59^. Several alleles were recently found under natural selection, likely due to the high-altitude lifestyle of this population^60^, including variants in the *EGLN1* (*HIF-PH2*) gene, which prevents degradation of hypoxia-inducible factors (HIFs). This HIF response allows for increased expression of hypoxia-inducible genes and confers a low hematocrit phenotype, which is advantageous in high altitudes and showed expression changes in the NASA Twins Study^6^. The Tibetan population has an enrichment of mutations in the *EPAS1* (*HIF-2A*) gene, which encodes subunits of HIFs that contribute to the development of blood vessels and render a low hemoglobin phenotype, while reducing any negative fitness consequences of excess red blood cell production^60^. Moreover, alleles in the *MTHFR* gene tend to create a low homocysteine and high folate phenotype in the Tibetan population, which can provide protection from hyperhomocysteinemia, endothelial cell injury, inflammation of blood vessels, and ischemic injury. The high folate phenotype also provides compensatory protection from UV radiation exposure, given that UV radiation degrades folate and folic acid in human blood.

Elucidating how the Bajau and Tibetan populations adapted to hypoxia may have clinical implications for people with conditions such as anemia, cytopenia, and hypoxia from intensive care treatment, tumorigenesis, and life in space. Also, carbon dioxide levels are typically higher on the ISS than on Earth and are associated with decreased cognitive scores and increased headaches in astronauts^6,61^. Furthermore, gas composition in future Martian settlements could deviate at even greater ranges, given the atmospheric differences and unknown habitat designs.

Humans have also experienced allelic adaptations to dietary habits, including lactase persistence from pastoralism in African populations^62^ and modulation of fatty acid metabolism from fatty acid-rich diets in Greenland populations^63^. Although these specific diets may not have direct implications for space nutrition, spaceflight food is restricted, and the diets of space travelers significantly differ from nominal terrestrial diets. Investigation of the interaction of dietary habits and omics may one day lead to healthier solutions for the limited spaceflight menu and possibly survey how future Martian societies biologically adapt over multiple generations to their unique nutritional lifestyles.

### Studying omics for improved spaceflight nutrition

Nutritional counseling based on omics has also benefited individuals with both rare (phenylketonuria) and common (lactose intolerance) genetic variants that entail customized dietary approaches^64^. Other evidence of genetic variants modifying responses to numerous nutrients and food bioactives has also emerged, including vitamins (A, B12, C, and D), fats (polyunsaturated and monounsaturated fatty acids), caffeine, calcium, choline, folate, and iron^65^. Stratified outcomes were found for immunity, inflammation, the health of various organs (bone, heart, muscle, and liver), endurance, strength training recovery, and visuomotor skills, all of which can be pivotal for athletes, the young, or the elderly.

Given the above evidence, omics metrics can also inform nutrition during space exploration. One unique example where this application has already proven meaningful relates to a distinctive ailment called spaceflight-associated neuro-ocular syndrome (SANS), which has no known terrestrial analogues. The disease appears to present in a subset of astronauts after short- and long-duration spaceflight, more so in men than women, and includes choroidal folds, optic disk edema (swelling), and focal areas of the ischemic retina (cotton wool spots). Research has suggested that SANS may be associated with lower levels of various types of vitamin B, combined with common variations in one-carbon metabolism genes^66^. This intriguing finding illustrates the potential utility of omics to inform optimal nutrition during spaceflight.

Dietary interventions specifically related to alleles listed in Fig. 3 could counteract space-induced inflammation. For example, several plant polyphenols can directly or indirectly activate *SIRT1*, which can reduce immune dysfunction, oxidative stress, autoimmunity, and inflammation^67^. In addition to immunological issues in space, the human body stores more iron during spaceflight (e.g. NASA Twins Study), which has been linked to increased oxidative stress and decreased regional BMD, further underscoring the potential of plant-based, antioxidant interventions for spaceflight nutrition. Also, improved insulin resistance from *SIRT1* may share common biochemical pathways with numerous foods rich in polyphenols, including resveratrol (peanuts, grapes, and berries); piceatannol (Japanese knotweed); and fisetin (lotus root, persimmons, and apples)^68^. Space crews often experience increased insulin resistance^69^ and thus may benefit from consuming some nutritional derivatives, particularly those who are prone to possess low levels of *SIRT1*. Studying numerous other space-related allele variants displayed in Fig. 3 may similarly inform aerospace nutrition: The polyphenol curcumin increases *LRP5* messenger RNA (mRNA) expression and may instill similar bone-protective mechanisms of *LRP5* variants^70^ as described above, the pigment β-Carotene is linked to the anti-aging benefits similar to *TERT* overproduction variants and may mitigate telomere attrition^71^, flavones have inhibitory effects on *MAFBX* (*FBXO32*, *ATROGIN-1*) expression and may prevent myotube diameter reduction during muscle atrophy^72^, and the plant alkaloid Berberine may inhibit *PCSK9* and reduce unhealthy cholesterol levels^73^.

Moreover, as astrobotany techniques progress, a wider selection of food crops could become successfully cultivable in space, and their nutritional and therapeutic properties could translate into valid countermeasures that avoid the negative side effects of pharmaceuticals and other space health approaches. Plant cells can deliver biomolecules that could benefit health in spaceflight: indeed, plants bioencapsulated with proinsulin have been shown to regulate blood sugar levels^74^. Metabolomics can help disentangle which space-farmed products may confer the most effective remedies for human illnesses during space exposure and which plants hold the greatest nutritional benefit per weight^75^. Plants in spaceflight can provide not only a wider assortment of nutrients, but also better air regeneration, water recycling, and mental health for astronauts, which could reduce flight risks. Also, discoveries in low-input and low-waste vegetation technology in space could be spun off to enhance sustainable agriculture back on Earth.

### Pharmacogenomics and optimized drug prescribing in space

Spaceflight pharmacogenomics (SPGx) is the study of gene variants that influence the regulation of drug metabolism in space operations^76,77^. The drug-gene response can influence safety and efficacy, which can have a notable influence on health and performance. To date, there are roughly 570 drug-gene combinations used in terrestrial medicine for which pharmacogenomics-guided prescribing recommendations have been established by the FDA.

One example of PGx relevant to spaceflight regards the phenomenon of statin-induced myopathy. Muscle loss in space remains a persistent challenge, so agents that facilitate myopathy in space are contraindicated. This can be contextualized by reviewing an investigation of a large military veteran cohort. Out of 7,769,356 veterans who took part in the study, nearly 55% took at least one drug with strong evidence of altered metabolism associated with a specific gene mutation over the six-year study period. Of particular concern, of the 533,928 participants who received new prescriptions of simvastatin, 25.6% were projected to have a SNP variant in *SLC01B1* (solute carrier organic anion transporter family member 1B1). The SLCO1B1*5 variant represents a loss‐of‐function allele known to attenuate the hepatic uptake of atorvastatin^78^. This allele results in an increase in the amount of the parent drug in systemic circulation, which may put individuals at risk for myopathy, no matter the dose.

The principle of PGx in space can be further illustrated by the case of codeine, which acts as a pain reliever via conversion to morphine by CYP450 2D6. Astronauts with the CYP450 2D6 slow metabolizer phenotype would be expected to convert only 10% of codeine to morphine. Therefore, codeine would be ineffective and should be removed from the mission drug list. Conversely, a 2D6 ultrarapid metabolizer will convert between 40 and 50% of codeine to morphine, which may result in morphine overdose and risk mission safety. In this case, codeine should be removed from the astronaut mission drug list because it is not safe^79,80^. Similar patterns have been seen for tramadol, where it may be ineffective for pain relief in slow metabolizers of CYP450 2D6. In 2D6 ultra-rapid metabolizers, the Dutch Pharmacogenetics Working Group recommends reducing tramadol dosage by 30% because of its tendency to produce symptoms such as nausea, vomiting, constipation, respiratory depression, confusion, and urinary retention^81^.

Each of the above symptoms would be unideal or unacceptable on a space mission. Fortunately, many drug-associated adverse events are predictable and repeatable, because of known drug-gene dynamics. Spaceflight PGx can be used to tailor mission drug lists in such a way that drug efficacy can be optimized, drug side effects can be reduced, and astronaut safety and performance enhanced.

### Technical challenges of studying human space omics

There are many challenges when studying genotype-phenotype interactions, both on Earth and in spaceflight. Engaging with national efforts (e.g. UK Biobank and the AllofUS program), as well as international groups like the International Standards for Space Omics Processing (ISSOP), can help develop standardization for space omics data and clinical applications^82^. These efforts can help optimize the extraction of actionable scientific findings from spaceflight omics data and increase the probability of safe and effective countermeasures in aerospace healthcare.

Alongside this promising side, we also follow with examples of the numerous technical and ethical difficulties involved with basic scientific research into the protective mechanisms linked to these alleles. For instance, the protective mechanisms associated with these alleles may present differently between in vitro and in vivo studies, study designs, and location (in space versus on Earth). These challenges are further convoluted by pleiotropy, whereby secondary effects may accompany or even outweigh protective effects. In addition, the small sample sizes in the human space omics discipline may also increase the risk of over-associating spaceflight omics changes with clinical meanings. Given these complexities, careful collection of standardized data and metadata; multi-omics profiling of consenting crew members before, during, and after spaceflight; and methodical production of cell space atlases could all be employed to better inform how to design safe and effective therapeutic compounds, meal plans, exercise equipment, and lifestyle recommendations that enhance protective effects and diminish harmful effects in space^83^.

### Statistical power, accuracy, and orthologs

Some difficulties with mapping protective alleles (e.g. in genome-wide association studies, GWAS) include the lack of replicability, non-coding annotations, and population stratification, which also are relevant for any polygenic risk score (PRS). The generalizability of a PRS can be poor if the population of interest does not align with the ancestry of the original GWAS study, a fact that can perpetuate health disparities^84^. Another difficulty relates to how evidence for genetic variations translates across populations, based on the impact of rare versus common variants and low effect sizes versus large effect sizes^85^. For example, the *MTHFR* gene is known to be highly polymorphic, and studies linking the A222V allele to diseases have found conflicting results based on ancestry^86^. Potential solutions may require increasing sample sizes of study populations and accounting for factors like geographical location, genome reference, ancestry, sex, and age^87^.

In addition, as shown in Supplementary Data 2, research into potentially protective alleles can sometimes have inconsistent findings between studies, and this may be partially due to experimental differences that must be clearly documented in metadata. The timeline of exposure is one of many metadata factors that can drastically change measured outcomes. Standardization of these metadata will be crucial to derive meaningful information from space omics studies^82^.

### Pleiotropic complications

Eliciting protective genotype-phenotype relationships is also challenging because several gene variants linked to protective phenotypes can concurrently also be linked to negative, potentially fatal, phenotypes. For example, while overproduction of *TERT* is linked to decreased aging, it is also linked to cancer risk^88^; while variants of *PCSK9* are linked to decreased LDL and heart disease^89^, they are also linked to diabetes^90^ and low cognition^91^; while *CCR5* variants are linked to decreased risks of cancer^92^, HIV^93^ and diseases of the heart^94^, liver^95^, and brain^96^, they also correspond to increased risks of various viruses like West Nile virus^97^; and while *CTNNB1* overproduction is linked to radiation resistance^98^, it is also linked to increased cancer risk^99^. To complicate the matter, downstream effects of gene variants can interact with each other; this is the case with *SOST* and *LRP5*, both related to bone health^100^. Any nutrition and lifestyle recommendations, and any compounds developed, from basic studies of these alleles would need to meticulously ensure that concomitant negative effects are circumvented.

Increasing the granularity of measures will allow for a better understanding of the cellular microenvironments and individual cellular subtypes, which could disentangle pleiotropic complications. Indeed, several papers referenced in Supplementary Data 2 emphasize the need for cell-type-specific studies to further resolve the physiology of the alleles^101^. Single cell sequencing technologies can generate high dimensional maps that resolve heterogeneous immunological cell types, allowing for better delineation of immune responses to various exposures and better interpretation of the immunosuppressive effects of spaceflight^102^.

One specific example is an overexpression of *SIRT1*, which could deliver protective phenotypes. However, *SIRT1* may increase the lifespan of rodent subjects when only overexpressed in particular regions, but not when overexpressed in the whole body^103,104^. Moreover, a global increase in the expression of *SIRT1* throughout all cells in the body may even confer negative effects, particularly within subset populations of T-helper cells, where it has been demonstrated to compromise differentiation. Hence, understanding optimal expression changes at the cell-type and lineage-specific level will be necessary to account for pleiotropic effects^105^.

### Space-relevant harmful alleles

We note that there are naturally occurring human alleles we did not highlight in this paper that are, in contrast, primarily known to be linked to space-related harmful effects. For example, mutations in the *ATM* gene may cause the disorder ataxia telangiectasia, and mutations in the *NBS1* gene may cause Nijmegen breakage syndrome; in both cases, affected individuals can show chromosomal instability, radiation sensitivity, and cancer susceptibility^106–108^. Additional genes related to DNA repair, radiation sensitivity, and cancer predisposition include *RAD50*, *BRCA1, BRCA2, P53*, among many others^109–111^. Investigating these alleles may guide risk reduction during space exploration, as well as for any type of experience on Earth in which individuals are exposed to higher-than-average amounts of radiation.

Another example is the I148M variant of the *PNPLA3* gene, associated with an increased risk of chronic liver disease^112^. Interestingly, harmful effects of the *PNPLA3* I148M mutation can be mitigated by one of the protective variants we listed in Fig. 3 (the rs72613567 variant of the *HSD17B13* gene) in an allele and dose-dependent manner^113^. Counteracting risk for liver disease could be important, since space stressors may activate lipotoxic pathways that further elevate liver disease risk, potentially leading to lipid dysregulation and reduced immune activity in susceptible spaceflight participants^114,115^.

In exploring genetic markers that confer potential risk in space, it is useful to further explore the APOE4/4 (APOE4) genotype in the context of novel exposures and cognitive changes. For example, there is a well-established phenomenon of chemotherapy-related cognitive impairment (CRCI), which appears to be increased in APOE4 carriers^116^. In survivors of breast cancer who had undergone chemotherapy, APOE4 carriers had worse visual memory and spatial ability when examined five years after diagnosis. In breast cancer survivors, those APOE4 carriers undergoing chemotherapy had significantly lower scores on measures of working memory and processing speed when examined at 1-, 6-, and 18-months post-treatment^117^. In another study, APOE4 breast cancer survivors treated with cytostatics had lower scores on executive function, attention, and processing speed at one- and two-years post-treatment^118^. Moreover, radiation-induced cognitive decline shows similar deficits in cognition. Cramer et al. showed that, among patients undergoing therapeutic brain irradiation, roughly 30% experience cognitive decline by 4 months. In those living over 6 months, the rate increases to 50% or greater^119^. Makale et al. have reported that 50-90% of those undergoing partial or whole brain cranial radiation experience disabling cognitive impairment (thought APOE status was not assessed by Cramer or Makale)^120^.

There is a paucity of human data related to APOE4 and spaceflight-associated cognitive risks. If the APOE4 genotype confers additional risk to cognitive decline in the presence of an exogenous stressor like space radiation, this would warrant careful attention to the APOE4 genotype in the future design of experiments concerning human spaceflight. It will also be important to determine whether the APOE2/2 genotype may be protective against cognitive decline in space in a manner similar to the protective effects of APOE2 on cognition on Earth.

To illustrate one final example, the R506Q mutation of the *F5* gene (Factor V Leiden) is one of the most common inherited susceptibilities to thrombophilia (blood clotting). The likelihood of blood clotting, which can be life-threatening, increases up to 7-fold in heterozygous carriers and up to 80-fold in homozygous individuals^121^. Immobility (such as during flight travel), increased estrogen levels (such as during pregnancy, hormone replacement therapy, and oral contraceptive use), and injuries (such as fractured bones) can further increase the risk for affected individuals^122^. Astronauts are frequently exposed to these associated risks, given the immobile nature of orbital flights, the personal decision by some participants to suppress menstruation with oral contraceptives during space missions, and the projected heightened risk of bone fractures in long-duration spaceflight^123^. Individuals could select specific countermeasures for blood clotting (such as compression stockings and anticoagulant substances) based on their personal risk profiles^124^.

### Ethical challenges of studying human space omics

As discussed in this paper, the landscape of human spaceflight has entered a transformative period of democratization, with increased commercial flight opportunities and collaborative agreements between government and private entities^125^. This increased diversity in mission formats, flight objectives, and stakeholders is creating a ripple effect on ethics and regulation. Indeed, many policy discussions in the context of human research and healthcare in space that were once internal to government institutions now fall under the remit of the public forum, as discussed elsewhere^126^. Widened discussion of policy has been further expedited by a push for open science, reflected through international data sharing and collaboration initiatives, including the NASA Open Science Data Repository^127^ and the EXPAND (Enhancing eXploration Platforms and Analog Definition) program of the Translational Research Institute for Space Health (TRISH)^128^.

Space agencies and commercial entities seek to identify biomarkers for spaceflight health effects to refine risk prediction, optimize mission planning, and elevate the standard of care for humans in space via improved countermeasures. In this paper, we have reviewed a number of published alleles naturally occurring in humans on Earth, and we have suggested their potential influence on the body’s response to the spaceflight environment. The use of allelic information for optimizing precision healthcare is commonplace on Earth, such as to avoid adverse reactions during drug prescriptions^129^, identify high-risk individuals to preemptively screen for disease onset, or refine diagnosis and inform therapeutic approach^130^. Similarly, allelic information and other omics could be used to optimize healthcare in space, such as through integration into precision healthcare strategies for government and commercial crew, the advent of which has been discussed elsewhere^131^. Of note, care should be taken that these alleles are not used for discriminatory practices, and updated legislation like the Genetic Information Non-Discrimination Act (GINA) may be needed to account for the new risk paradigm of spaceflight and long-term care or life insurance.

In terrestrial healthcare, emergent omics approaches also include genetic and epigenetic modification therapies^132^. If safely established on Earth, in the far future, one can imagine that similar modification therapies may eventually be investigated as candidate interventions for increased health and safety in crewed spaceflight, such as to protect against space stressors during long-duration missions to Mars, the outer solar system, and even beyond, as recently discussed^18,105^. While beyond the scope of this manuscript, we note that gene therapy is a particularly controversial subject matter, and its associated ethical challenges have been discussed elsewhere in the context of human spaceflight^133,134^.

## Discussion

In the foreseeable future, collecting, storing, and sharing omics data from consenting humans venturing into space for use in research and terrestrially established occupational healthcare strategies also requires careful policy to address a multitude of ethical challenges. Indeed, ethical challenges include developing data accessibility and sharing strategies without violation of privacy of individuals and family members, achieving meaningful informed consent throughout the entire data lifecycle, avoiding uses of data that support actions of a discriminatory or inequitable nature, ensuring thorough and expert ethical oversight of projects, and appropriately handling any incidental findings from the data^134^, as well as issues regarding the challenge of protecting astronauts^135,136^. Just as the landscape of human spaceflight is diversifying in a positive and more inclusive manner, so too are the opportunities for improving precision healthcare in flight and on longer missions^137^. For example, with genetic risk data in hand, individuals could exercise increased autonomy in making informed decisions about participating in spaceflight activities.

In this paper, we reviewed an example set of published alleles purportedly linked to protective effects, the biochemical pathways of which could inform therapeutic compound discovery and nutrition, exercise, and lifestyle recommendations to protect health during spaceflight missions and potentially during postflight recovery and stationing on planetary surfaces, including the Moon and Mars^138^. This guide can also help individuals on Earth in high-stress and environmentally challenging scenarios, such as first responders, cave explorers, radiation workers, deep-sea investigators, or commercial astronauts like the Inspiration4, Axoim Space, and Polaris Dawn missions^91,139–147^. Alongside any untapped potential to enhance both terrestrial and aerospace precision healthcare, investigation of such alleles poses significant technical and ethical challenges. If humanity safely and effectively surmounts these hurdles, the revamped aerospace health recommendations that ensue could be incorporated with other types of countermeasures (such as artificial gravity, faster spacecraft, radiation shielding, and biotechnology for food and material production) in order to best preserve the well-being of life during future deep space missions and interplanetary exploration.

### Supplementary information

Description of Additional Supplementary Files

Supplementary Data 1

Supplementary Data 2

Supplementary Data 1 References

Supplementary Data 2 References
