# Supplementary material for: Protective alleles and precision healthcare in crewed spaceflight
Source: Nat Commun. 2024 Jul 22;15:6158. doi: 10.1038/s41467-024-49423-6 (PMC11263583; doi:10.1038/s41467-024-49423-6)
Supplement: Supplementary file 1 — Description of Additional Supplementary Files [file 41467_2024_49423_MOESM1_ESM.docx]

Supplementary Data Legends for

“Protective alleles and precision healthcare in crewed spaceflight”

Authors: Lindsay A. Rutter, Matthew J. MacKay, Henry Cope, Nathaniel J. Szewczyk, JangKeun

Kim, Eliah Overbey, Braden T. Tierney, Masafumi Muratani, Ben Lamm, Daniela Bezdan,

Amber M. Paul, Michael A. Schmidt, George M. Church, Stefania Giacomello, Christopher E.

Mason

Supplementary Table 1

Examples of health-risk categories that warrant precision countermeasures to improve the

physical fitness of both spaceflight participants and terrestrial humankind, complete with

references. From left to right: examples of common spaceflight ailments related to the health-

risk category; examples of spaceflight countermeasures related to the health-risk category;

examples of terrestrial populations that could benefit from improved precision countermeasures

related to the health-risk category.

Supplementary Table 2

Literature review and its key findings for a subset of alleles linked to protective effects, many of

which occur naturally in the human population. From left to right: the health category; the gene

name; the allele in rodents and humans; example protective effects; example harmful effects;

example papers showing protective effects at various levels of evidence (in vivo or in vitro, on

Earth or in space, in humans or in rodents); a brief explanation of the primary mode of study for

each example paper; a basic description of how the wild type gene functions on Earth;

examples of how the space environment may affect the gene function; a basic description of

how the protective allele may function; example clinical trials that target the pathway of the

protective allele; examples of lifestyles (exercise and diet) that target the pathway of the

protective allele. Empty cells indicate instances in which we were unable to find moderate

evidence based on current literature. This table was manually curated and prone to our

subjective interpretations. Literature references are provided for readers to review more details.
