# Supplementary material for: Protective alleles and precision healthcare in crewed spaceflight
Source: Nat Commun. 2024 Jul 22;15:6158. doi: 10.1038/s41467-024-49423-6 (PMC11263583; doi:10.1038/s41467-024-49423-6)
Supplement: Supplementary file 4 — Supplementary Data 1 References [file 41467_2024_49423_MOESM4_ESM.docx]

1. [Garrett-Bakelman FE, Darshi M, Green SJ, Gur RC, Lin L, Macias BR, et al. The NASA Twins Study: A multidimensional analysis of a year-long human spaceflight. Science. 2019;364. doi:](http://paperpile.com/b/GLFHrG/FRTYn)[10.1126/science.aau8650](http://dx.doi.org/10.1126/science.aau8650)

2. [Shammas MA. Telomeres, lifestyle, cancer, and aging. Current Opinion in Clinical Nutrition and Metabolic Care. 2011. pp. 28–34. doi:](http://paperpile.com/b/GLFHrG/XgyPZ)[10.1097/mco.0b013e32834121b1](http://dx.doi.org/10.1097/mco.0b013e32834121b1)

3. [Lee J-Y, Jun N-R, Yoon D, Shin C, Baik I. Association between dietary patterns in the remote past and telomere length. Eur J Clin Nutr. 2015;69: 1048–1052.](http://paperpile.com/b/GLFHrG/0S0dg)

4. [Lang TF, Leblanc AD, Evans HJ, Lu Y. Adaptation of the proximal femur to skeletal reloading after long-duration spaceflight. J Bone Miner Res. 2006;21: 1224–1230.](http://paperpile.com/b/GLFHrG/nrQYP)

5. [Rea R, Beck C, Rovekamp R, Neuhaus P, Diftler M. X1: A Robotic Exoskeleton for In-Space Countermeasures and Dynamometry. AIAA SPACE 2013 Conference and Exposition. 2013. doi:](http://paperpile.com/b/GLFHrG/mpYFQ)[10.2514/6.2013-5510](http://dx.doi.org/10.2514/6.2013-5510)

6. [Shiba D, Mizuno H, Yumoto A, Shimomura M, Kobayashi H, Morita H, et al. Development of new experimental platform “MARS”-Multiple Artificial-gravity Research System-to elucidate the impacts of micro/partial gravity on mice. Sci Rep. 2017;7: 10837.](http://paperpile.com/b/GLFHrG/W8tBg)

7. [Leblanc A, Matsumoto T, Jones J, Shapiro J, Lang T, Shackelford L, et al. Bisphosphonates as a supplement to exercise to protect bone during long-duration spaceflight. Osteoporos Int. 2013;24: 2105–2114.](http://paperpile.com/b/GLFHrG/w7A4O)

8. [Smith S, Heer M, Zwart S. Nutrition and Human Space Flight: Evidence from 4–6 Month Missions to the International Space Station. Current Developments in Nutrition. 2020. pp. 1146–1146. doi:](http://paperpile.com/b/GLFHrG/Gith0)[10.1093/cdn/nzaa055_031](http://dx.doi.org/10.1093/cdn/nzaa055_031)

9. [Abrams SA, Griffin IJ, Hawthorne KM, Liang L, Gunn SK, Darlington G, et al. A combination of prebiotic short- and long-chain inulin-type fructans enhances calcium absorption and bone mineralization in young adolescents. The American Journal of Clinical Nutrition. 2005. pp. 471–476. doi:](http://paperpile.com/b/GLFHrG/W5HEZ)[10.1093/ajcn/82.2.471](http://dx.doi.org/10.1093/ajcn/82.2.471)

10. [Smith SM, Heer MA, Shackelford LC, Sibonga JD, Ploutz-Snyder L, Zwart SR. Benefits for bone from resistance exercise and nutrition in long-duration spaceflight: Evidence from biochemistry and densitometry. J Bone Miner Res. 2012;27: 1896–1906.](http://paperpile.com/b/GLFHrG/Zqpde)

11. [Baca J, Martinez M, Coles S, Garcia A, Berthelot D, McCumber J, et al. Modular multi-motor exercise system for space exploration. SN Applied Sciences. 2020. doi:](http://paperpile.com/b/GLFHrG/M6PfE)[10.1007/s42452-020-2315-1](http://dx.doi.org/10.1007/s42452-020-2315-1)

12. [Cortese F, Klokov D, Osipov A, Stefaniak J, Moskalev A, Schastnaya J, et al. Vive la radiorésistance!: converging research in radiobiology and biogerontology to enhance human radioresistance for deep space exploration and colonization. Oncotarget. 2018;9: 14692–14722.](http://paperpile.com/b/GLFHrG/wr3GQ)

13. [Stein TP, Schulter MD, Boden G. Development of insulin resistance by astronauts during spaceflight. Aviat Space Environ Med. 1994;65: 1091–1096.](http://paperpile.com/b/GLFHrG/Yg0Dz)

14. [Tobin BW, Uchakin PN, Leeper-Woodford SK. Insulin secretion and sensitivity in space flight: diabetogenic effects. Nutrition. 2002;18: 842–848.](http://paperpile.com/b/GLFHrG/lDyp6)

15. [Levin DR, Blue RS, Castleberry TL, Vanderploeg JM. Tolerance of centrifuge-simulated suborbital spaceflight in subjects with implanted insulin pumps. Aerosp Med Hum Perform. 2015;86: 407–409.](http://paperpile.com/b/GLFHrG/RyUjE)

16. [Gallo C, Ridolfi L, Scarsoglio S. Cardiovascular deconditioning during long-term spaceflight through multiscale modeling. NPJ Microgravity. 2020;6: 27.](http://paperpile.com/b/GLFHrG/ytMC9)

17. [Tuday EC, Meck JV, Nyhan D, Shoukas AA, Berkowitz DE. Microgravity-induced changes in aortic stiffness and their role in orthostatic intolerance. J Appl Physiol. 2007;102: 853–858.](http://paperpile.com/b/GLFHrG/6FSY9)

18. [Meerman M, Bracco Gartner TCL, Buikema JW, Wu SM, Siddiqi S, Bouten CVC, et al. Myocardial Disease and Long-Distance Space Travel: Solving the Radiation Problem. Front Cardiovasc Med. 2021;8: 631985.](http://paperpile.com/b/GLFHrG/CYUId)

19. [Peters SAE, Bots SH, Woodward M. Sex Differences in the Association Between Measures of General and Central Adiposity and the Risk of Myocardial Infarction: Results From the UK Biobank. J Am Heart Assoc. 2018;7. doi:](http://paperpile.com/b/GLFHrG/SWkm4)[10.1161/JAHA.117.008507](http://dx.doi.org/10.1161/JAHA.117.008507)

20. [Wewege MA, Ahn D, Yu J, Liou K, Keech A. High‐Intensity Interval Training for Patients With Cardiovascular Disease—Is It Safe? A Systematic Review. Journal of the American Heart Association. 2018. doi:](http://paperpile.com/b/GLFHrG/SxAyB)[10.1161/jaha.118.009305](http://dx.doi.org/10.1161/jaha.118.009305)

21. [Rooney BV, Crucian BE, Pierson DL, Laudenslager ML, Mehta SK. Herpes Virus Reactivation in Astronauts During Spaceflight and Its Application on Earth. Front Microbiol. 2019;10: 16.](http://paperpile.com/b/GLFHrG/zllsq)

22. [Taylor PW. Impact of space flight on bacterial virulence and antibiotic susceptibility. Infect Drug Resist. 2015;8: 249–262.](http://paperpile.com/b/GLFHrG/EIzdI)

23. [Nickerson CA, Ott CM, Mister SJ, Morrow BJ, Burns-Keliher L, Pierson DL. Microgravity as a novel environmental signal affecting Salmonella enterica serovar Typhimurium virulence. Infect Immun. 2000;68: 3147–3152.](http://paperpile.com/b/GLFHrG/qh9So)

24. [Ott CM, Bruce RJ, Pierson DL. Microbial characterization of free floating condensate aboard the Mir space station. Microb Ecol. 2004;47: 133–136.](http://paperpile.com/b/GLFHrG/I5l83)

25. [Crucian B, Stowe R, Mehta S, Uchakin P, Quiriarte H, Pierson D, et al. Immune system dysregulation occurs during short duration spaceflight on board the space shuttle. J Clin Immunol. 2013;33: 456–465.](http://paperpile.com/b/GLFHrG/rBdjW)

26. [Akiyama T, Horie K, Hinoi E, Hiraiwa M, Kato A, Maekawa Y, et al. How does spaceflight affect the acquired immune system? NPJ Microgravity. 2020;6: 14.](http://paperpile.com/b/GLFHrG/f3BIu)

27. [Simpson RJ, Kunz H, Agha N, Graff R. Exercise and the Regulation of Immune Functions. Prog Mol Biol Transl Sci. 2015;135: 355–380.](http://paperpile.com/b/GLFHrG/8fQbZ)

28. [Agha NH, Mehta SK, Rooney BV, Laughlin MS, Markofski MM, Pierson DL, et al. Exercise as a countermeasure for latent viral reactivation during long duration space flight. FASEB J. 2020;34: 2869–2881.](http://paperpile.com/b/GLFHrG/QBZjw)

29. [Douglas GL, Voorhies AA. Evidence based selection of probiotic strains to promote astronaut health or alleviate symptoms of illness on long duration spaceflight missions. Benef Microbes. 2017;8: 727–737.](http://paperpile.com/b/GLFHrG/xtyVW)

30. [Chung S, Yao H, Caito S, Hwang J-W, Arunachalam G, Rahman I. Regulation of SIRT1 in cellular functions: role of polyphenols. Arch Biochem Biophys. 2010;501: 79–90.](http://paperpile.com/b/GLFHrG/cDdwN)

31. [Glaser R, Litsky ML, Padgett DA, Baiocchi RA, Yang EV, Chen M, et al. EBV-encoded dUTPase induces immune dysregulation: Implications for the pathophysiology of EBV-associated disease. Virology. 2006;346: 205–218.](http://paperpile.com/b/GLFHrG/F709r)

32. [Castanon-Cervantes O, Wu M, Ehlen JC, Paul K, Gamble KL, Johnson RL, et al. Dysregulation of inflammatory responses by chronic circadian disruption. J Immunol. 2010;185: 5796–5805.](http://paperpile.com/b/GLFHrG/N9rzT)

33. [Wong WC, Oubre C, Mehta SK, Mark Ott C, Pierson DL. Preventing Infectious Diseases in Spacecraft and Space Habitats. Modeling the Transmission and Prevention of Infectious Disease. 2017. pp. 3–17. doi:](http://paperpile.com/b/GLFHrG/TLbJk)[10.1007/978-3-319-60616-3_1](http://dx.doi.org/10.1007/978-3-319-60616-3_1)

34. [Cain JR. Astronaut Health—Planetary Exploration and the Limitations on Freedom. The Meaning of Liberty Beyond Earth. 2015. pp. 139–163. doi:](http://paperpile.com/b/GLFHrG/SLqNm)[10.1007/978-3-319-09567-7_10](http://dx.doi.org/10.1007/978-3-319-09567-7_10)

35. [Checinska A, Probst AJ, Vaishampayan P, White JR, Kumar D, Stepanov VG, et al. Microbiomes of the dust particles collected from the International Space Station and Spacecraft Assembly Facilities. Microbiome. 2015;3: 50.](http://paperpile.com/b/GLFHrG/lV7ZS)

36. [Wong WC, Dudinsky LA, Garcia VM, Ott CM, Castro VA. Efficacy of various chemical disinfectants on biofilms formed in spacecraft potable water system components. Biofouling. 2010;26: 583–586.](http://paperpile.com/b/GLFHrG/xjkUY)

37. [Kim HW, Rhee MS. Space food and bacterial infections: Realities of the risk and role of science. Trends in Food Science & Technology. 2020. pp. 275–287. doi:](http://paperpile.com/b/GLFHrG/pIISq)[10.1016/j.tifs.2020.10.023](http://dx.doi.org/10.1016/j.tifs.2020.10.023)

38. [Douglas GL, Massa GD, Hummerick ME, Hintze PE. Cold plasma to disinfect spaceflight grown produce. Advances in Cold Plasma Applications for Food Safety and Preservation. 2020. pp. 333–340. doi:](http://paperpile.com/b/GLFHrG/0EMPy)[10.1016/b978-0-12-814921-8.00012-8](http://dx.doi.org/10.1016/b978-0-12-814921-8.00012-8)

39. [Bashir M, Ahmed M, Weinmaier T, Ciobanu D, Ivanova N, Pieber TR, et al. Functional Metagenomics of Spacecraft Assembly Cleanrooms: Presence of Virulence Factors Associated with Human Pathogens. Front Microbiol. 2016;7: 1321.](http://paperpile.com/b/GLFHrG/wO9og)

40. [Beheshti A, Chakravarty K, Fogle H, Fazelinia H, Silveira WA da, Boyko V, et al. Author Correction: Multi-omics analysis of multiple missions to space reveal a theme of lipid dysregulation in mouse liver. Sci Rep. 2020;10: 1517.](http://paperpile.com/b/GLFHrG/CzSs9)

41. [Jonscher KR, Alfonso-Garcia A, Suhalim JL, Orlicky DJ, Potma EO, Ferguson VL, et al. Spaceflight Activates Lipotoxic Pathways in Mouse Liver. PLoS One. 2016;11: e0152877.](http://paperpile.com/b/GLFHrG/35cFP)

42. [Romero-Gómez M, Zelber-Sagi S, Trenell M. Treatment of NAFLD with diet, physical activity and exercise. J Hepatol. 2017;67: 829–846.](http://paperpile.com/b/GLFHrG/MRxmh)

43. [Vandenburgh H, Chromiak J, Shansky J, Del Tatto M, Lemaire J. Space travel directly induces skeletal muscle atrophy. FASEB J. 1999;13: 1031–1038.](http://paperpile.com/b/GLFHrG/TFRVR)

44. [Kozlovskaya IB, Grigoriev AI, Stepantzov VI. Countermeasure of the negative effects of weightlessness on physical systems in long-term space flights. Acta Astronaut. 1995;36: 661–668.](http://paperpile.com/b/GLFHrG/tNGPH)

45. [Lathers CM, Charles JB. Use of lower body negative pressure to counter symptoms of orthostatic intolerance in patients, bed rest subjects, and astronauts. J Clin Pharmacol. 1993;33: 1071–1085.](http://paperpile.com/b/GLFHrG/YBLzg)

46. [Clément GR, Bukley AP, Paloski WH. Artificial gravity as a countermeasure for mitigating physiological deconditioning during long-duration space missions. Front Syst Neurosci. 2015;9: 92.](http://paperpile.com/b/GLFHrG/7rZdB)

47. [Law J, Van Baalen M, Foy M, Mason SS, Mendez C, Wear ML, et al. Relationship Between Carbon Dioxide Levels and Reported Headaches on the International Space Station. Journal of Occupational and Environmental Medicine. 2014. pp. 477–483. doi:](http://paperpile.com/b/GLFHrG/ueCLf)[10.1097/jom.0000000000000158](http://dx.doi.org/10.1097/jom.0000000000000158)

48. [Conkin J, Wessel JH 3rd, Norcross JR, Bekdash OS, Abercromby AFJ, Koslovsky MD, et al. Hemoglobin Oxygen Saturation with Mild Hypoxia and Microgravity. Aerosp Med Hum Perform. 2017;88: 527–534.](http://paperpile.com/b/GLFHrG/tlj0G)

49. [Pagnini F, Phillips D, Bercovitz K, Langer E. Mindfulness and relaxation training for long duration spaceflight: Evidences from analog environments and military settings. Acta Astronautica. 2019. pp. 1–8. doi:](http://paperpile.com/b/GLFHrG/gkSd9)[10.1016/j.actaastro.2019.07.036](http://dx.doi.org/10.1016/j.actaastro.2019.07.036)

50. [Chancellor JC, Blue RS, Cengel KA, Auñón-Chancellor SM, Rubins KH, Katzgraber HG, et al. Limitations in predicting the space radiation health risk for exploration astronauts. NPJ Microgravity. 2018;4: 8.](http://paperpile.com/b/GLFHrG/u7LzE)

51. [Hopewell JW. Models of CNS radiation damage during space flight. Adv Space Res. 1994;14: 433–442.](http://paperpile.com/b/GLFHrG/smFqe)

52. [Vasileiou T, Summerer L. Correction: A biomimetic approach to shielding from ionizing radiation: The case of melanized fungi. PLoS One. 2021;16: e0257068.](http://paperpile.com/b/GLFHrG/7AwmV)

53. [Narici L, Casolino M, Di Fino L, Larosa M, Picozza P, Rizzo A, et al. Performances of Kevlar and Polyethylene as radiation shielding on-board the International Space Station in high latitude radiation environment. Sci Rep. 2017;7: 1644.](http://paperpile.com/b/GLFHrG/pMZKG)

54. [Mortazavi SMJ, Cameron JR, Niroomand-Rad A. The life saving role of radioadaptive responses in long-term interplanetary space journeys. International Congress Series. 2005. pp. 266–267. doi:](http://paperpile.com/b/GLFHrG/Bb0W4)[10.1016/j.ics.2004.12.019](http://dx.doi.org/10.1016/j.ics.2004.12.019)

55. [Blakely WF, Miller AC, Grace MB, McLeland CB, Luo L, Muderhwa JM, et al. Radiation biodosimetry: applications for spaceflight. Adv Space Res. 2003;31: 1487–1493.](http://paperpile.com/b/GLFHrG/n8N4L)

56. [Beheshti A, Miller J, Kidane Y, Berrios D, Gebre SG, Costes SV. NASA GeneLab Project: Bridging Space Radiation Omics with Ground Studies. Radiation Research. 2018. pp. 553–559. doi:](http://paperpile.com/b/GLFHrG/PviwI)[10.1667/rr15062.1](http://dx.doi.org/10.1667/rr15062.1)

57. [Smith SM, Zwart SR. Chapter 3 Nutritional Biochemistry Of Spaceflight. Advances in Clinical Chemistry. 2008. pp. 87–130. doi:](http://paperpile.com/b/GLFHrG/PtzB5)[10.1016/s0065-2423(08)00403-4](http://dx.doi.org/10.1016/s0065-2423(08)00403-4)

58. [Hu S, Kim M-HY, McClellan GE, Cucinotta FA. Modeling the acute health effects of astronauts from exposure to large solar particle events. Health Phys. 2009;96: 465–476.](http://paperpile.com/b/GLFHrG/M6mwn)

59. [Barger LK, Flynn-Evans EE, Kubey A, Walsh L, Ronda JM, Wang W, et al. Prevalence of sleep deficiency and use of hypnotic drugs in astronauts before, during, and after spaceflight: an observational study. Lancet Neurol. 2014;13: 904–912.](http://paperpile.com/b/GLFHrG/2FAcv)

60. [Mallis MM, DeRoshia CW. Circadian rhythms, sleep, and performance in space. Aviat Space Environ Med. 2005;76: B94–107.](http://paperpile.com/b/GLFHrG/zEEq4)

61. [Wotring VE. Medication use by U.S. crewmembers on the International Space Station. FASEB J. 2015;29: 4417–4423.](http://paperpile.com/b/GLFHrG/tnxMM)
