# Supplementary material for: Protective alleles and precision healthcare in crewed spaceflight
Source: Nat Commun. 2024 Jul 22;15:6158. doi: 10.1038/s41467-024-49423-6 (PMC11263583; doi:10.1038/s41467-024-49423-6)
Supplement: Supplementary file 5 — Supplementary Data 2 References [file 41467_2024_49423_MOESM5_ESM.docx]

1. [Tomás-Loba A, Flores I, Fernández-Marcos PJ, Cayuela ML, Maraver A, Tejera A, et al. Telomerase reverse transcriptase delays aging in cancer-resistant mice. Cell. 2008;135: 609–622.](http://paperpile.com/b/6W2Kpw/sdLb)

2. [Kim NW, Piatyszek MA, Prowse KR, Harley CB, West MD, Ho PL, et al. Specific association of human telomerase activity with immortal cells and cancer. Science. 1994;266: 2011–2015.](http://paperpile.com/b/6W2Kpw/30dj)

3. [Artandi SE, Alson S, Tietze MK, Sharpless NE, Ye S, Greenberg RA, et al. Constitutive telomerase expression promotes mammary carcinomas in aging mice. Proc Natl Acad Sci U S A. 2002;99: 8191–8196.](http://paperpile.com/b/6W2Kpw/139L)

4. [Bernardes de Jesus B, Vera E, Schneeberger K, Tejera AM, Ayuso E, Bosch F, et al. Telomerase gene therapy in adult and old mice delays aging and increases longevity without increasing cancer. EMBO Mol Med. 2012;4: 691–704.](http://paperpile.com/b/6W2Kpw/8N0k)

5. [Berardinelli F, Antoccia A, Buonsante R, Gerardi S, Cherubini R, De Nadal V, et al. The role of telomere length modulation in delayed chromosome instability induced by ionizing radiation in human primary fibroblasts. Environ Mol Mutagen. 2013;54: 172–179.](http://paperpile.com/b/6W2Kpw/Rphe)

6. [Sishc BJ, Nelson CB, McKenna MJ, Battaglia CLR, Herndon A, Idate R, et al. Telomeres and Telomerase in the Radiation Response: Implications for Instability, Reprograming, and Carcinogenesis. Frontiers in Oncology. 2015. doi:](http://paperpile.com/b/6W2Kpw/f20j)[10.3389/fonc.2015.00257](http://dx.doi.org/10.3389/fonc.2015.00257)

7. [Luxton JJ, McKenna MJ, Taylor LE, George KA, Zwart SR, Crucian BE, et al. Temporal Telomere and DNA Damage Responses in the Space Radiation Environment. Cell Rep. 2020;33: 108435.](http://paperpile.com/b/6W2Kpw/d4NR)

8. [Garrett-Bakelman FE, Darshi M, Green SJ, Gur RC, Lin L, Macias BR, et al. The NASA Twins Study: A multidimensional analysis of a year-long human spaceflight. Science. 2019;364. doi:](http://paperpile.com/b/6W2Kpw/sdQo)[10.1126/science.aau8650](http://dx.doi.org/10.1126/science.aau8650)

9. [Shammas MA. Telomeres, lifestyle, cancer, and aging. Current Opinion in Clinical Nutrition and Metabolic Care. 2011. pp. 28–34. doi:](http://paperpile.com/b/6W2Kpw/i2Cj)[10.1097/mco.0b013e32834121b1](http://dx.doi.org/10.1097/mco.0b013e32834121b1)

10. [Babij P, Zhao W, Small C, Kharode Y, Yaworsky PJ, Bouxsein ML, et al. High bone mass in mice expressing a mutant LRP5 gene. J Bone Miner Res. 2003;18: 960–974.](http://paperpile.com/b/6W2Kpw/iR09)

11. [Little RD, Folz C, Manning SP, Swain PM, Zhao S-C, Eustace B, et al. A Mutation in the LDL Receptor–Related Protein 5 Gene Results in the Autosomal Dominant High–Bone-Mass Trait. The American Journal of Human Genetics. 2002. pp. 11–19. doi:](http://paperpile.com/b/6W2Kpw/XMVF)[10.1086/338450](http://dx.doi.org/10.1086/338450)

12. [Zhang Y, Wang Y, Li X, Zhang J, Mao J, Li Z, et al. The LRP5 high-bone-mass G171V mutation disrupts LRP5 interaction with Mesd. Mol Cell Biol. 2004;24: 4677–4684.](http://paperpile.com/b/6W2Kpw/ZMku)

13. [Saxon LK, Jackson BF, Sugiyama T, Lanyon LE, Price JS. Analysis of multiple bone responses to graded strains above functional levels, and to disuse, in mice in vivo show that the human Lrp5 G171V High Bone Mass mutation increases the osteogenic response to loading but that lack of Lrp5 activity reduces it. Bone. 2011;49: 184–193.](http://paperpile.com/b/6W2Kpw/RR2q)

14. [Williams BO, Insogna KL. Where Wnts went: the exploding field of Lrp5 and Lrp6 signaling in bone. J Bone Miner Res. 2009;24: 171–178.](http://paperpile.com/b/6W2Kpw/cJ2z)

15. [Qin W, Zhao W, Li X, Peng Y, Harlow LM, Li J, et al. Mice with sclerostin gene deletion are resistant to the severe sublesional bone loss induced by spinal cord injury. Osteoporos Int. 2016;27: 3627–3636.](http://paperpile.com/b/6W2Kpw/ADr1)

16. [Recker RR, Benson CT, Matsumoto T, Bolognese MA, Robins DA, Alam J, et al. A randomized, double-blind phase 2 clinical trial of blosozumab, a sclerostin antibody, in postmenopausal women with low bone mineral density. J Bone Miner Res. 2015;30: 216–224.](http://paperpile.com/b/6W2Kpw/cREG)

17. [Spatz JM, Wein MN, Gooi JH, Qu Y, Garr JL, Liu S, et al. The Wnt Inhibitor Sclerostin Is Up-regulated by Mechanical Unloading in Osteocytes in Vitro. J Biol Chem. 2015;290: 16744–16758.](http://paperpile.com/b/6W2Kpw/j5U8)

18. [Macaulay TR, Siamwala JH, Hargens AR, Macias BR. Thirty days of spaceflight does not alter murine calvariae structure despite increased Sost expression. Bone Reports. 2017. pp. 57–62. doi:](http://paperpile.com/b/6W2Kpw/FbZq)[10.1016/j.bonr.2017.08.004](http://dx.doi.org/10.1016/j.bonr.2017.08.004)

19. [Spatz JM, Fields EE, Yu EW, Divieti Pajevic P, Bouxsein ML, Sibonga JD, et al. Serum sclerostin increases in healthy adult men during bed rest. J Clin Endocrinol Metab. 2012;97: E1736–40.](http://paperpile.com/b/6W2Kpw/ZY2U)

20. [Li X, Zhang Y, Kang H, Liu W, Liu P, Zhang J, et al. Sclerostin Binds to LRP5/6 and Antagonizes Canonical Wnt Signaling. Journal of Biological Chemistry. 2005. pp. 19883–19887. doi:](http://paperpile.com/b/6W2Kpw/Tdzu)[10.1074/jbc.m413274200](http://dx.doi.org/10.1074/jbc.m413274200)

21. [Cosman F, Crittenden DB, Adachi JD, Binkley N, Czerwinski E, Ferrari S, et al. Romosozumab Treatment in Postmenopausal Women with Osteoporosis. N Engl J Med. 2016;375: 1532–1543.](http://paperpile.com/b/6W2Kpw/qT6z)

22. [Macias BR, Swift JM, Nilsson MI, Hogan HA, Bouse SD, Bloomfield SA. Simulated resistance training, but not alendronate, increases cortical bone formation and suppresses sclerostin during disuse. J Appl Physiol. 2012;112: 918–925.](http://paperpile.com/b/6W2Kpw/cZTE)

23. [Coschigano KT, Holland AN, Riders ME, List EO, Flyvbjerg A, Kopchick JJ. Deletion, But Not Antagonism, of the Mouse Growth Hormone Receptor Results in Severely Decreased Body Weights, Insulin, and Insulin-Like Growth Factor I Levels and Increased Life Span. Endocrinology. 2003. pp. 3799–3810. doi:](http://paperpile.com/b/6W2Kpw/cj0Y)[10.1210/en.2003-0374](http://dx.doi.org/10.1210/en.2003-0374)

24. [Rosenbloom AL, Aguirre JG, Rosenfeld RG, Fielder PJ. The Little Women of Loja — Growth Hormone–Receptor Deficiency in an Inbred Population of Southern Ecuador. New England Journal of Medicine. 1990. pp. 1367–1374. doi:](http://paperpile.com/b/6W2Kpw/TV29)[10.1056/nejm199011153232002](http://dx.doi.org/10.1056/nejm199011153232002)

25. [Guevara-Aguirre J, Balasubramanian P, Guevara-Aguirre M, Wei M, Madia F, Cheng C-W, et al. Growth hormone receptor deficiency is associated with a major reduction in pro-aging signaling, cancer, and diabetes in humans. Sci Transl Med. 2011;3: 70ra13.](http://paperpile.com/b/6W2Kpw/hWWv)

26. [Berryman DE, List EO, Coschigano KT, Behar K, Kim JK, Kopchick JJ. Comparing adiposity profiles in three mouse models with altered GH signaling. Growth Horm IGF Res. 2004;14: 309–318.](http://paperpile.com/b/6W2Kpw/3pmx)

27. [Wu X, Wan M, Li G, Xu Z, Chen C, Liu F, et al. Growth hormone receptor overexpression predicts response of rectal cancers to pre-operative radiotherapy. Eur J Cancer. 2006;42: 888–894.](http://paperpile.com/b/6W2Kpw/Td7j)

28. [McCall GE, Goulet C, Roy RR, Grindeland RE, Boorman GI, Bigbee AJ, et al. Spaceflight suppresses exercise-induced release of bioassayable growth hormone. J Appl Physiol. 1999;87: 1207–1212.](http://paperpile.com/b/6W2Kpw/SChh)

29. [McCall GE, Goulet C, Grindeland RE, Hodgson JA, Bigbee AJ, Edgerton VR. Bed rest suppresses bioassayable growth hormone release in response to muscle activity. Journal of Applied Physiology. 1997. pp. 2086–2090. doi:](http://paperpile.com/b/6W2Kpw/1XGg)[10.1152/jappl.1997.83.6.2086](http://dx.doi.org/10.1152/jappl.1997.83.6.2086)

30. [Dehkhoda F, Lee CMM, Medina J, Brooks AJ. The Growth Hormone Receptor: Mechanism of Receptor Activation, Cell Signaling, and Physiological Aspects. Front Endocrinol . 2018;9: 35.](http://paperpile.com/b/6W2Kpw/I1Qa)

31. [Evans A, Jamieson SMF, Liu D-X, Wilson WR, Perry JK. Growth hormone receptor antagonism suppresses tumour regrowth after radiotherapy in an endometrial cancer xenograft model. Cancer Letters. 2016. pp. 117–123. doi:](http://paperpile.com/b/6W2Kpw/iaX9)[10.1016/j.canlet.2016.05.031](http://dx.doi.org/10.1016/j.canlet.2016.05.031)

32. [Wideman L, Weltman JY, Hartman ML, Veldhuis JD, Weltman A. Growth hormone release during acute and chronic aerobic and resistance exercise: recent findings. Sports Med. 2002;32: 987–1004.](http://paperpile.com/b/6W2Kpw/x0Bi)

33. [Clasey JL, Weltman A, Patrie J, Weltman JY, Pezzoli S, Bouchard C, et al. Abdominal visceral fat and fasting insulin are important predictors of 24-hour GH release independent of age, gender, and other physiological factors. J Clin Endocrinol Metab. 2001;86: 3845–3852.](http://paperpile.com/b/6W2Kpw/yDps)

34. [Matheu A, Pantoja C, Efeyan A, Criado LM, Martín-Caballero J, Flores JM, et al. Increased gene dosage of Ink4a/Arf results in cancer resistance and normal aging. Genes Dev. 2004;18: 2736–2746.](http://paperpile.com/b/6W2Kpw/6sTO)

35. [de Snoo FA, Hayward NK. Cutaneous melanoma susceptibility and progression genes. Cancer Lett. 2005;230: 153–186.](http://paperpile.com/b/6W2Kpw/Wc2Q)

36. [Laderian B, Fojo T. CDK4/6 Inhibition as a therapeutic strategy in breast cancer: palbociclib, ribociclib, and abemaciclib. Semin Oncol. 2017;44: 395–403.](http://paperpile.com/b/6W2Kpw/6K3d)

37. [Sun C, Zhang F, Ge X, Yan T, Chen X, Shi X, et al. SIRT1 improves insulin sensitivity under insulin-resistant conditions by repressing PTP1B. Cell Metab. 2007;6: 307–319.](http://paperpile.com/b/6W2Kpw/EZlC)

38. [Shen P, Deng X, Chen Z, Ba X, Qin K, Huang Y, et al. SIRT1: A Potential Therapeutic Target in Autoimmune Diseases. Front Immunol. 2021;12: 779177.](http://paperpile.com/b/6W2Kpw/6i7O)

39. [Liu L, Cheng Y, Wang J, Ding Z, Halim A, Luo Q, et al. Simulated Microgravity Suppresses Osteogenic Differentiation of Mesenchymal Stem Cells by Inhibiting Oxidative Phosphorylation. Int J Mol Sci. 2020;21. doi:](http://paperpile.com/b/6W2Kpw/ewwL)[10.3390/ijms21249747](http://dx.doi.org/10.3390/ijms21249747)

40. [Cao Y, Jiang X, Ma H, Wang Y, Xue P, Liu Y. SIRT1 and insulin resistance. Journal of Diabetes and its Complications. 2016. pp. 178–183. doi:](http://paperpile.com/b/6W2Kpw/fmoV)[10.1016/j.jdiacomp.2015.08.022](http://dx.doi.org/10.1016/j.jdiacomp.2015.08.022)

41. [Yoshizaki T, Schenk S, Imamura T, Babendure JL, Sonoda N, Bae EJ, et al. SIRT1 inhibits inflammatory pathways in macrophages and modulates insulin sensitivity. Am J Physiol Endocrinol Metab. 2010;298: E419–28.](http://paperpile.com/b/6W2Kpw/qJWi)

42. [Kawakami S, Kinoshita Y, Maruki-Uchida H, Yanae K, Sai M, Ito T. Piceatannol and its metabolite, isorhapontigenin, induce SIRT1 expression in THP-1 human monocytic cell line. Nutrients. 2014;6: 4794–4804.](http://paperpile.com/b/6W2Kpw/M2ln)

43. [Hwang ES, Song SB. Nicotinamide is an inhibitor of SIRT1 in vitro, but can be a stimulator in cells. Cell Mol Life Sci. 2017;74: 3347–3362.](http://paperpile.com/b/6W2Kpw/J1pH)

44. [Vargas-Ortiz K, Pérez-Vázquez V, Macías-Cervantes MH. Exercise and Sirtuins: A Way to Mitochondrial Health in Skeletal Muscle. Int J Mol Sci. 2019;20. doi:](http://paperpile.com/b/6W2Kpw/kDDx)[10.3390/ijms20112717](http://dx.doi.org/10.3390/ijms20112717)

45. [Li D, Weng S, Yang B, Zander DS, Saldeen T, Nichols WW, et al. Inhibition of arterial thrombus formation by ApoA1 Milano. Arterioscler Thromb Vasc Biol. 1999;19: 378–383.](http://paperpile.com/b/6W2Kpw/MSWN)

46. [Gualandri V, Franceschini G, Sirtori CR, Gianfranceschi G, Orsini GB, Cerrone A, et al. AIMilano apoprotein identification of the complete kindred and evidence of a dominant genetic transmission. Am J Hum Genet. 1985;37: 1083–1097.](http://paperpile.com/b/6W2Kpw/l1XE)

47. [Nissen SE, Tsunoda T, Tuzcu EM, Schoenhagen P, Cooper CJ, Yasin M, et al. Effect of recombinant ApoA-I Milano on coronary atherosclerosis in patients with acute coronary syndromes: a randomized controlled trial. JAMA. 2003;290: 2292–2300.](http://paperpile.com/b/6W2Kpw/duXC)

48. [Chistiakov DA, Bobryshev YV, Orekhov AN. Macrophage-mediated cholesterol handling in atherosclerosis. J Cell Mol Med. 2016;20: 17–28.](http://paperpile.com/b/6W2Kpw/Ruuh)

49. [Franceschini G, Sirtori CR, Capurso A, Weisgraber KH, Mahley RW. A-IMilano apoprotein. Decreased high density lipoprotein cholesterol levels with significant lipoprotein modifications and without clinical atherosclerosis in an Italian family. Journal of Clinical Investigation. 1980. pp. 892–900. doi:](http://paperpile.com/b/6W2Kpw/QctD)[10.1172/jci109956](http://dx.doi.org/10.1172/jci109956)

50. [Rashid S, Curtis DE, Garuti R, Anderson NN, Bashmakov Y, Ho YK, et al. Decreased plasma cholesterol and hypersensitivity to statins in mice lacking Pcsk9. Proc Natl Acad Sci U S A. 2005;102: 5374–5379.](http://paperpile.com/b/6W2Kpw/AG7Z)

51. [Cohen JC, Boerwinkle E, Mosley TH Jr, Hobbs HH. Sequence variations in PCSK9, low LDL, and protection against coronary heart disease. N Engl J Med. 2006;354: 1264–1272.](http://paperpile.com/b/6W2Kpw/vrxN)

52. [Schmidt AF, Swerdlow DI, Holmes MV, Patel RS, Fairhurst-Hunter Z, Lyall DM, et al. PCSK9 genetic variants and risk of type 2 diabetes: a mendelian randomisation study. Lancet Diabetes Endocrinol. 2017;5: 97–105.](http://paperpile.com/b/6W2Kpw/wZbk)

53. [Mannarino MR, Sahebkar A, Bianconi V, Serban M-C, Banach M, Pirro M. PCSK9 and neurocognitive function: Should it be still an issue after FOURIER and EBBINGHAUS results? J Clin Lipidol. 2018;12: 1123–1132.](http://paperpile.com/b/6W2Kpw/3dsr)

54. [Park SW, Moon Y-A, Horton JD. Post-transcriptional regulation of low density lipoprotein receptor protein by proprotein convertase subtilisin/kexin type 9a in mouse liver. J Biol Chem. 2004;279: 50630–50638.](http://paperpile.com/b/6W2Kpw/Y0Ai)

55. [Schwartz GG, Steg PG, Szarek M, Bhatt DL, Bittner VA, Diaz R, et al. Alirocumab and Cardiovascular Outcomes after Acute Coronary Syndrome. N Engl J Med. 2018;379: 2097–2107.](http://paperpile.com/b/6W2Kpw/sGru)

56. [Sabatine MS, Giugliano RP, Keech AC, Honarpour N, Wiviott SD, Murphy SA, et al. Evolocumab and Clinical Outcomes in Patients with Cardiovascular Disease. New England Journal of Medicine. 2017. pp. 1713–1722. doi:](http://paperpile.com/b/6W2Kpw/yShG)[10.1056/nejmoa1615664](http://dx.doi.org/10.1056/nejmoa1615664)

57. [Adorni MP, Zimetti F, Lupo MG, Ruscica M, Ferri N. Naturally Occurring PCSK9 Inhibitors. Nutrients. 2020;12. doi:](http://paperpile.com/b/6W2Kpw/4ejW)[10.3390/nu12051440](http://dx.doi.org/10.3390/nu12051440)

58. [Levenson AE, Milliren CE, Biddinger SB, Ebbeling CB, Feldman HA, Ludwig DS, et al. Calorically restricted diets decrease PCSK9 in overweight adolescents. Nutr Metab Cardiovasc Dis. 2017;27: 342–349.](http://paperpile.com/b/6W2Kpw/dEcm)

59. [Dewey FE, Gusarova V, Dunbar RL, O’Dushlaine C, Schurmann C, Gottesman O, et al. Genetic and Pharmacologic Inactivation of ANGPTL3 and Cardiovascular Disease. N Engl J Med. 2017;377: 211–221.](http://paperpile.com/b/6W2Kpw/xP2h)

60. [Rudwill F, Bergouignan A, Gastebois C, Gauquelin-Koch G, Lefai E, Blanc S, et al. Effect of enforced physical inactivity induced by 60-day of bed rest on hepatic markers of NAFLD in healthy normal-weight women. Liver Int. 2015;35: 1700–1706.](http://paperpile.com/b/6W2Kpw/EoX8)

61. [Tikka A, Jauhiainen M. The role of ANGPTL3 in controlling lipoprotein metabolism. Endocrine. 2016;52: 187–193.](http://paperpile.com/b/6W2Kpw/8Fg3)

62. [Butler AA, Graham JL, Stanhope KL, Wong S, King S, Bremer AA, et al. Role of angiopoietin-like protein 3 in sugar-induced dyslipidemia in rhesus macaques: suppression by fish oil or RNAi. Journal of Lipid Research. 2020. pp. 376–386. doi:](http://paperpile.com/b/6W2Kpw/ofYP)[10.1194/jlr.ra119000423](http://dx.doi.org/10.1194/jlr.ra119000423)

63. [Kaplan R, Zhang T, Hernandez M, Gan FX, Wright SD, Waters MG, et al. Regulation of the angiopoietin-like protein 3 gene by LXR. J Lipid Res. 2003;44: 136–143.](http://paperpile.com/b/6W2Kpw/Yr8b)

64. [Zhou M, Greenhill S, Huang S, Silva TK, Sano Y, Wu S, et al. CCR5 is a suppressor for cortical plasticity and hippocampal learning and memory. Elife. 2016;5. doi:](http://paperpile.com/b/6W2Kpw/OTxS)[10.7554/eLife.20985](http://dx.doi.org/10.7554/eLife.20985)

65. [Hütter G, Nowak D, Mossner M, Ganepola S, Müssig A, Allers K, et al. Long-term control of HIV by CCR5 Delta32/Delta32 stem-cell transplantation. N Engl J Med. 2009;360: 692–698.](http://paperpile.com/b/6W2Kpw/Xac6)

66. [Joy MT, Ben Assayag E, Shabashov-Stone D, Liraz-Zaltsman S, Mazzitelli J, Arenas M, et al. CCR5 Is a Therapeutic Target for Recovery after Stroke and Traumatic Brain Injury. Cell. 2019;176: 1143–1157.e13.](http://paperpile.com/b/6W2Kpw/PdAY)

67. [Lin C-S, Hsieh P-S, Hwang L-L, Lee Y-H, Tsai S-H, Tu Y-C, et al. The CCL5/CCR5 Axis Promotes Vascular Smooth Muscle Cell Proliferation and Atherogenic Phenotype Switching. Cell Physiol Biochem. 2018;47: 707–720.](http://paperpile.com/b/6W2Kpw/gOZR)

68. [Perez-Martinez L, Perez-Matute P, Aguilera-Lizarraga J, Rubio-Mediavilla S, Narro J, Recio E, et al. Maraviroc, a CCR5 antagonist, ameliorates the development of hepatic steatosis in a mouse model of non-alcoholic fatty liver disease (NAFLD). Journal of Antimicrobial Chemotherapy. 2014. pp. 1903–1910. doi:](http://paperpile.com/b/6W2Kpw/EXcS)[10.1093/jac/dku071](http://dx.doi.org/10.1093/jac/dku071)

69. [Halama N, Zoernig I, Berthel A, Kahlert C, Klupp F, Suarez-Carmona M, et al. Tumoral Immune Cell Exploitation in Colorectal Cancer Metastases Can Be Targeted Effectively by Anti-CCR5 Therapy in Cancer Patients. Cancer Cell. 2016;29: 587–601.](http://paperpile.com/b/6W2Kpw/S9d8)

70. [Klein RS. A moving target: the multiple roles of CCR5 in infectious diseases. The Journal of infectious diseases. 2008. pp. 183–186.](http://paperpile.com/b/6W2Kpw/qbGr)

71. [Falcon A, Cuevas MT, Rodriguez-Frandsen A, Reyes N, Pozo F, Moreno S, et al. CCR5 deficiency predisposes to fatal outcome in influenza virus infection. J Gen Virol. 2015;96: 2074–2078.](http://paperpile.com/b/6W2Kpw/QFCm)

72. [Glass WG, McDermott DH, Lim JK, Lekhong S, Yu SF, Frank WA, et al. CCR5 deficiency increases risk of symptomatic West Nile virus infection. J Exp Med. 2006;203: 35–40.](http://paperpile.com/b/6W2Kpw/bmBr)

73. [Velasco-Velázquez M, Jiao X, De La Fuente M, Pestell TG, Ertel A, Lisanti MP, et al. CCR5 antagonist blocks metastasis of basal breast cancer cells. Cancer Res. 2012;72: 3839–3850.](http://paperpile.com/b/6W2Kpw/K1uM)

74. [Liu R, Paxton WA, Choe S, Ceradini D, Martin SR, Horuk R, et al. Homozygous defect in HIV-1 coreceptor accounts for resistance of some multiply-exposed individuals to HIV-1 infection. Cell. 1996;86: 367–377.](http://paperpile.com/b/6W2Kpw/HZTn)

75. [Benkirane M, Jin DY, Chun RF, Koup RA, Jeang KT. Mechanism of transdominant inhibition of CCR5-mediated HIV-1 infection by ccr5delta32. J Biol Chem. 1997;272: 30603–30606.](http://paperpile.com/b/6W2Kpw/tn2m)

76. [Nair MP, Kandaswami C, Mahajan S, Nair HN, Chawda R, Shanahan T, et al. Grape seed extract proanthocyanidins downregulate HIV-1 entry coreceptors, CCR2b, CCR3 and CCR5 gene expression by normal peripheral blood mononuclear cells. Biol Res. 2002;35: 421–431.](http://paperpile.com/b/6W2Kpw/cd8S)

77. [McPherron AC, Lawler AM, Lee S-J. Regulation of skeletal muscle mass in mice by a new TGF-p superfamily member. Nature. 1997. pp. 83–90. doi:](http://paperpile.com/b/6W2Kpw/tJXD)[10.1038/387083a0](http://dx.doi.org/10.1038/387083a0)

78. [Lee S-J, Lehar A, Meir JU, Koch C, Morgan A, Warren LE, et al. Targeting myostatin/activin A protects against skeletal muscle and bone loss during spaceflight. Proc Natl Acad Sci U S A. 2020;117: 23942–23951.](http://paperpile.com/b/6W2Kpw/igXR)

79. [McMahon CD, Popovic L, Oldham JM, Jeanplong F, Smith HK, Kambadur R, et al. Myostatin-deficient mice lose more skeletal muscle mass than wild-type controls during hindlimb suspension. Am J Physiol Endocrinol Metab. 2003;285: E82–7.](http://paperpile.com/b/6W2Kpw/sp49)

80. [Dankbar B, Fennen M, Brunert D, Hayer S, Frank S, Wehmeyer C, et al. Myostatin is a direct regulator of osteoclast differentiation and its inhibition reduces inflammatory joint destruction in mice. Nat Med. 2015;21: 1085–1090.](http://paperpile.com/b/6W2Kpw/Kd4z)

81. [Schuelke M, Wagner KR, Stolz LE, Hübner C, Riebel T, Kömen W, et al. Myostatin Mutation Associated with Gross Muscle Hypertrophy in a Child. New England Journal of Medicine. 2004. pp. 2682–2688. doi:](http://paperpile.com/b/6W2Kpw/am9s)[10.1056/nejmoa040933](http://dx.doi.org/10.1056/nejmoa040933)

82. [Jugdutt BI, Dhalla NS. Cardiac Remodeling: Molecular Mechanisms. Springer Science & Business Media; 2013.](http://paperpile.com/b/6W2Kpw/VZCo)

83. [Smith RC, Cramer MS, Mitchell PJ, Lucchesi J, Ortega AM, Livingston EW, et al. Inhibition of myostatin prevents microgravity-induced loss of skeletal muscle mass and strength. PLoS One. 2020;15: e0230818.](http://paperpile.com/b/6W2Kpw/1JSE)

84. [Irimia JM, Guerrero M, Rodriguez-Miguelez P, Cadefau JA, Tesch PA, Cussó R, et al. Metabolic adaptations in skeletal muscle after 84 days of bed rest with and without concurrent flywheel resistance exercise. J Appl Physiol. 2017;122: 96–103.](http://paperpile.com/b/6W2Kpw/D6IY)

85. [Becker C, Lord SR, Studenski SA, Warden SJ, Fielding RA, Recknor CP, et al. Myostatin antibody (LY2495655) in older weak fallers: a proof-of-concept, randomised, phase 2 trial. Lancet Diabetes Endocrinol. 2015;3: 948–957.](http://paperpile.com/b/6W2Kpw/Y0QU)

86. [Saremi A, Gharakhanloo R, Sharghi S, Gharaati MR, Larijani B, Omidfar K. Effects of oral creatine and resistance training on serum myostatin and GASP-1. Mol Cell Endocrinol. 2010;317: 25–30.](http://paperpile.com/b/6W2Kpw/Vmj1)

87. [Adam M, Heikelä H, Sobolewski C, Portius D, Mäki-Jouppila J, Mehmood A, et al. Hydroxysteroid (17β) dehydrogenase 13 deficiency triggers hepatic steatosis and inflammation in mice. The FASEB Journal. 2018. pp. 3434–3447. doi:](http://paperpile.com/b/6W2Kpw/rMDh)[10.1096/fj.201700914r](http://dx.doi.org/10.1096/fj.201700914r)

88. [Abul-Husn NS, Cheng X, Li AH, Xin Y, Schurmann C, Stevis P, et al. A Protein-Truncating HSD17B13 Variant and Protection from Chronic Liver Disease. N Engl J Med. 2018;378: 1096–1106.](http://paperpile.com/b/6W2Kpw/jq0S)

89. [Ma Y, Belyaeva OV, Brown PM, Fujita K, Valles K, Karki S, et al. 17-Beta Hydroxysteroid Dehydrogenase 13 Is a Hepatic Retinol Dehydrogenase Associated With Histological Features of Nonalcoholic Fatty Liver Disease. Hepatology. 2019;69: 1504–1519.](http://paperpile.com/b/6W2Kpw/WFB1)

90. [Kozlitina J, Stender S, Hobbs HH, Cohen JC. HSD17B13 and Chronic Liver Disease in Blacks and Hispanics. N Engl J Med. 2018;379: 1876–1877.](http://paperpile.com/b/6W2Kpw/Q0mX)

91. [Ma Y, Brown PM, Lin DD, Ma J, Feng D, Belyaeva OV, et al. 17‐Beta Hydroxysteroid Dehydrogenase 13 Deficiency Does Not Protect Mice From Obesogenic Diet Injury. Hepatology. 2021. pp. 1701–1716. doi:](http://paperpile.com/b/6W2Kpw/nwxe)[10.1002/hep.31517](http://dx.doi.org/10.1002/hep.31517)

92. [Obajemu AA, Rao N, Dilley KA, Vargas JM, Sheikh F, Donnelly RP, et al. IFN-λ4 Attenuates Antiviral Responses by Enhancing Negative Regulation of IFN Signaling. J Immunol. 2017;199: 3808–3820.](http://paperpile.com/b/6W2Kpw/6Wmj)

93. [Prokunina-Olsson L, Muchmore B, Tang W, Pfeiffer RM, Park H, Dickensheets H, et al. A variant upstream of IFNL3 (IL28B) creating a new interferon gene IFNL4 is associated with impaired clearance of hepatitis C virus. Nat Genet. 2013;45: 164–171.](http://paperpile.com/b/6W2Kpw/5g93)

94. [Real LM, Herrero R, Rivero-Juárez A, Camacho Á, Macías J, Vic S, et al. IFNL4 rs368234815 polymorphism is associated with innate resistance to HIV-1 infection. AIDS. 2015;29: 1895–1897.](http://paperpile.com/b/6W2Kpw/a3ZP)

95. [Prokunina-Olsson L, Morrison RD, Obajemu A, Mahamar A, Kim S, Attaher O, et al. IFN-λ4 is associated with increased risk and earlier occurrence of several common infections in African children. Genes Immun. 2021;22: 44–55.](http://paperpile.com/b/6W2Kpw/KOfH)

96. [Rahimi P, Tarharoudi R, Rahimpour A, Mosayebi Amroabadi J, Ahmadi I, Anvari E, et al. The association between interferon lambda 3 and 4 gene single-nucleotide polymorphisms and the recovery of COVID-19 patients. Virol J. 2021;18: 221.](http://paperpile.com/b/6W2Kpw/G1mC)

97. [Jenkins FJ, Minas TZ, Tang W, Dorsey TH, Ambs S. Human herpesvirus 8 infection is associated with prostate cancer among IFNL4-ΔG carriers. Prostate Cancer Prostatic Dis. 2022. doi:](http://paperpile.com/b/6W2Kpw/deax)[10.1038/s41391-022-00546-1](http://dx.doi.org/10.1038/s41391-022-00546-1)

98. [Manuel O, Wójtowicz A, Bibert S, Mueller NJ, van Delden C, Hirsch HH, et al. Influence of IFNL3/4 polymorphisms on the incidence of cytomegalovirus infection after solid-organ transplantation. J Infect Dis. 2015;211: 906–914.](http://paperpile.com/b/6W2Kpw/10l6)

99. [Petta S, Valenti L, Tuttolomondo A, Dongiovanni P, Pipitone RM, Cammà C, et al. Interferon lambda 4 rs368234815 TT>δG variant is associated with liver damage in patients with nonalcoholic fatty liver disease. Hepatology. 2017;66: 1885–1893.](http://paperpile.com/b/6W2Kpw/ScG8)

100. [Key FM, Peter B, Dennis MY, Huerta-Sánchez E, Tang W, Prokunina-Olsson L, et al. Selection on a variant associated with improved viral clearance drives local, adaptive pseudogenization of interferon lambda 4 (IFNL4). PLoS Genet. 2014;10: e1004681.](http://paperpile.com/b/6W2Kpw/7IxQ)

101. [Conjeevaram HS, Fried MW, Jeffers LJ, Terrault NA, Wiley-Lucas TE, Afdhal N, et al. Peginterferon and ribavirin treatment in African American and Caucasian American patients with hepatitis C genotype 1. Gastroenterology. 2006;131: 470–477.](http://paperpile.com/b/6W2Kpw/UlZB)

102. [Bacon BR, Gordon SC, Lawitz E, Marcellin P, Vierling JM, Zeuzem S, et al. Boceprevir for Previously Treated Chronic HCV Genotype 1 Infection. New England Journal of Medicine. 2011. pp. 1207–1217. doi:](http://paperpile.com/b/6W2Kpw/lMor)[10.1056/nejmoa1009482](http://dx.doi.org/10.1056/nejmoa1009482)

103. [Muir AJ, Bornstein JD, Killenberg PG, Atlantic Coast Hepatitis Treatment Group. Peginterferon alfa-2b and ribavirin for the treatment of chronic hepatitis C in blacks and non-Hispanic whites. N Engl J Med. 2004;350: 2265–2271.](http://paperpile.com/b/6W2Kpw/sN1H)

104. [Bodine SC, Latres E, Baumhueter S, Lai VK, Nunez L, Clarke BA, et al. Identification of ubiquitin ligases required for skeletal muscle atrophy. Science. 2001;294: 1704–1708.](http://paperpile.com/b/6W2Kpw/qUQU)

105. [Neutelings T, Nusgens BV, Liu Y, Tavella S, Ruggiu A, Cancedda R, et al. Skin physiology in microgravity: a 3-month stay aboard ISS induces dermal atrophy and affects cutaneous muscle and hair follicles cycling in mice. NPJ Microgravity. 2015;1: 15002.](http://paperpile.com/b/6W2Kpw/RUyx)

106. [Sandonà D, Desaphy J-F, Camerino GM, Bianchini E, Ciciliot S, Danieli-Betto D, et al. Adaptation of mouse skeletal muscle to long-term microgravity in the MDS mission. PLoS One. 2012;7: e33232.](http://paperpile.com/b/6W2Kpw/Hoyy)

107. [Brooks NE, Cadena SM, Vannier E, Cloutier G, Carambula S, Myburgh KH, et al. Effects of resistance exercise combined with essential amino acid supplementation and energy deficit on markers of skeletal muscle atrophy and regeneration during bed rest and active recovery. Muscle Nerve. 2010;42: 927–935.](http://paperpile.com/b/6W2Kpw/U7DT)

108. [Shiota C, Abe T, Kawai N, Ohno A, Teshima-Kondo S, Mori H, et al. Flavones Inhibit LPS-Induced Atrogin-1/MAFbx Expression in Mouse C2C12 Skeletal Myotubes. J Nutr Sci Vitaminol . 2015;61: 188–194.](http://paperpile.com/b/6W2Kpw/CM4H)

109. [Salazar-Mendiguchía J, Ochoa JP, Palomino-Doza J, Domínguez F, Díez-López C, Akhtar M, et al. Mutations in cause an autosomal-recessive form of hypertrophic cardiomyopathy. Heart. 2020;106: 1342–1348.](http://paperpile.com/b/6W2Kpw/u0V2)

110. [Cadena SM, Zhang Y, Fang J, Brachat S, Kuss P, Giorgetti E, et al. Skeletal muscle in MuRF1 null mice is not spared in low-gravity conditions, indicating atrophy proceeds by unique mechanisms in space. Sci Rep. 2019;9: 9397.](http://paperpile.com/b/6W2Kpw/MWsp)

111. [Centner T, Yano J, Kimura E, McElhinny AS, Pelin K, Witt CC, et al. Identification of muscle specific ring finger proteins as potential regulators of the titin kinase domain. J Mol Biol. 2001;306: 717–726.](http://paperpile.com/b/6W2Kpw/lcLP)

112. [Zhang Y, Li Y, Hu Q, Xi Y, Xing Z, Zhang Z, et al. The lncRNA H19 alleviates muscular dystrophy by stabilizing dystrophin. Nat Cell Biol. 2020;22: 1332–1345.](http://paperpile.com/b/6W2Kpw/WwEs)

113. [Musarò A, Dobrowolny G, Cambieri C, Onesti E, Ceccanti M, Frasca V, et al. Neuromuscular magnetic stimulation counteracts muscle decline in ALS patients: results of a randomized, double-blind, controlled study. Sci Rep. 2019;9: 2837.](http://paperpile.com/b/6W2Kpw/QUad)

114. [Ilardo MA, Moltke I, Korneliussen TS, Cheng J, Stern AJ, Racimo F, et al. Physiological and Genetic Adaptations to Diving in Sea Nomads. Cell. 2018;173: 569–580.e15.](http://paperpile.com/b/6W2Kpw/JLj9)

115. [Rex DAB, Vaid N, Deepak K, Dagamajalu S, Prasad TSK. A comprehensive review on current understanding of bradykinin in COVID-19 and inflammatory diseases. Mol Biol Rep. 2022;49: 9915–9927.](http://paperpile.com/b/6W2Kpw/JKKU)

116. [Baranova TI, Berlov DN, Glotov OS, Korf EA, Minigalin AD, Mitrofanova AV, et al. Genetic determination of the vascular reactions in humans in response to the diving reflex. Am J Physiol Heart Circ Physiol. 2017;312: H622–H631.](http://paperpile.com/b/6W2Kpw/O80V)

117. [Gislén A, Warrant EJ, Dacke M, Kröger RHH. Visual training improves underwater vision in children. Vision Res. 2006;46: 3443–3450.](http://paperpile.com/b/6W2Kpw/MgtS)

118. [Yang J, Jin Z-B, Chen J, Huang X-F, Li X-M, Liang Y-B, et al. Genetic signatures of high-altitude adaptation in Tibetans. Proc Natl Acad Sci U S A. 2017;114: 4189–4194.](http://paperpile.com/b/6W2Kpw/HGZs)

119. [Scheinfeldt LB, Tishkoff SA. Living the high life: high-altitude adaptation. Genome Biol. 2010;11: 133.](http://paperpile.com/b/6W2Kpw/Lojk)

120. [Xia G, Kageyama Y, Hayashi T, Kawakami S, Yoshida M, Kihara K. Regulation of vascular endothelial growth factor transcription by endothelial PAS domain protein 1 (EPAS1) and possible involvement of EPAS1 in the angiogenesis of renal cell carcinoma. Cancer. 2001;91: 1429–1436.](http://paperpile.com/b/6W2Kpw/utei)

121. [Beall CM, Cavalleri GL, Deng L, Elston RC, Gao Y, Knight J, et al. Natural selection on EPAS1 (HIF2alpha) associated with low hemoglobin concentration in Tibetan highlanders. Proc Natl Acad Sci U S A. 2010;107: 11459–11464.](http://paperpile.com/b/6W2Kpw/c0KR)

122. [Botto LD, Yang Q. 5,10-Methylenetetrahydrofolate reductase gene variants and congenital anomalies: a HuGE review. Am J Epidemiol. 2000;151: 862–877.](http://paperpile.com/b/6W2Kpw/AK5X)

123. [Lewis SJ, Lawlor DA, Davey Smith G, Araya R, Timpson N, Day INM, et al. The thermolabile variant of MTHFR is associated with depression in the British Women’s Heart and Health Study and a meta-analysis. Mol Psychiatry. 2006;11: 352–360.](http://paperpile.com/b/6W2Kpw/NKLw)

124. [Schmidt RJ, Hansen RL, Hartiala J, Allayee H, Schmidt LC, Tancredi DJ, et al. Prenatal vitamins, one-carbon metabolism gene variants, and risk for autism. Epidemiology. 2011;22: 476–485.](http://paperpile.com/b/6W2Kpw/LZiY)

125. [Luo Z, Lu Z, Muhammad I, Chen Y, Chen Q, Zhang J, et al. Associations of the MTHFR rs1801133 polymorphism with coronary artery disease and lipid levels: a systematic review and updated meta-analysis. Lipids in Health and Disease. 2018. doi:](http://paperpile.com/b/6W2Kpw/wYBa)[10.1186/s12944-018-0837-y](http://dx.doi.org/10.1186/s12944-018-0837-y)

126. [Ojha RP, Gurney JG. Methylenetetrahydrofolate reductase C677T and overall survival in pediatric acute lymphoblastic leukemia: a systematic review. Leuk Lymphoma. 2014;55: 67–73.](http://paperpile.com/b/6W2Kpw/PQNI)

127. [de la Chapelle A, Träskelin AL, Juvonen E. Truncated erythropoietin receptor causes dominantly inherited benign human erythrocytosis. Proc Natl Acad Sci U S A. 1993;90: 4495–4499.](http://paperpile.com/b/6W2Kpw/fSiV)

128. [Israels LG, Israels ED. Erythropoiesis: an overview. Erythropoietins and Erythropoiesis. pp. 3–14. doi:](http://paperpile.com/b/6W2Kpw/UUNh)[10.1007/3-7643-7543-4_1](http://dx.doi.org/10.1007/3-7643-7543-4_1)

129. [Woodward WA, Chen MS, Behbod F, Alfaro MP, Buchholz TA, Rosen JM. WNT/beta-catenin mediates radiation resistance of mouse mammary progenitor cells. Proceedings of the National Academy of Sciences. 2007. pp. 618–623. doi:](http://paperpile.com/b/6W2Kpw/9y26)[10.1073/pnas.0606599104](http://dx.doi.org/10.1073/pnas.0606599104)

130. [Kim PJ, Plescia J, Clevers H, Fearon ER, Altieri DC. Survivin and molecular pathogenesis of colorectal cancer. Lancet. 2003;362: 205–209.](http://paperpile.com/b/6W2Kpw/5X9A)

131. [He H, Lin K, Su Y, Lin S, Zou C, Pan J, et al. Overexpression of β-Catenin Decreases the Radiosensitivity of Human Nasopharyngeal Carcinoma CNE-2 Cells. Cell Physiol Biochem. 2018;50: 1929–1944.](http://paperpile.com/b/6W2Kpw/xsxW)

132. [Liu X, Wang L, Zhao S, Ji X, Luo Y, Ling F. β-Catenin overexpression in malignant glioma and its role in proliferation and apoptosis in glioblastma cells. Med Oncol. 2011;28: 608–614.](http://paperpile.com/b/6W2Kpw/Li4S)

133. [Morin PJ. β-catenin signaling and cancer. BioEssays. 1999. pp. 1021–1030. doi:](http://paperpile.com/b/6W2Kpw/3wEP)[10.1002/(sici)1521-1878(199912)22:1<1021::aid-bies6>3.0.co;2-p](http://dx.doi.org/10.1002/(sici)1521-1878(199912)22:1%3C1021::aid-bies6%3E3.0.co;2-p)

134. [Mitra D, Luo X, Morgan A, Wang J, Hoang MP, Lo J, et al. An ultraviolet-radiation-independent pathway to melanoma carcinogenesis in the red hair/fair skin background. Nature. 2012;491: 449–453.](http://paperpile.com/b/6W2Kpw/s4vX)

135. [Swope V, Alexander C, Starner R, Schwemberger S, Babcock G, Abdel-Malek ZA. Significance of the melanocortin 1 receptor in the DNA damage response of human melanocytes to ultraviolet radiation. Pigment Cell Melanoma Res. 2014;27: 601–610.](http://paperpile.com/b/6W2Kpw/PYVX)

136. [Kadekaro AL, Chen J, Yang J, Chen S, Jameson J, Swope VB, et al. Alpha-melanocyte-stimulating hormone suppresses oxidative stress through a p53-mediated signaling pathway in human melanocytes. Mol Cancer Res. 2012;10: 778–786.](http://paperpile.com/b/6W2Kpw/x2GN)

137. [Funasaka Y, Chakraborty AK, Hayashi Y, Komoto M, Ohashi A, Nagahama M, et al. Modulation of melanocyte-stimulating hormone receptor expression on normal human melanocytes: evidence for a regulatory role of ultraviolet B, interleukin-1alpha, interleukin-1beta, endothelin-1 and tumour necrosis factor-alpha. Br J Dermatol. 1998;139: 216–224.](http://paperpile.com/b/6W2Kpw/rHlA)

138. [Corre S, Mekideche K, Adamski H, Mosser J, Watier E, Galibert M-D. In vivo and ex vivo UV-induced analysis of pigmentation gene expressions. J Invest Dermatol. 2006;126: 916–918.](http://paperpile.com/b/6W2Kpw/QAke)

139. [Rees JL, Flanagan N. Pigmentation, melanocortins and red hair. QJM. 1999;92: 125–131.](http://paperpile.com/b/6W2Kpw/08cq)

140. [Buscà R, Ballotti R. Cyclic AMP a key messenger in the regulation of skin pigmentation. Pigment Cell Res. 2000;13: 60–69.](http://paperpile.com/b/6W2Kpw/sK1z)

141. [Chakraborty AK, Orlow SJ, Bolognia JL, Pawelek JM. Structural/functional relationships between internal and external MSH receptors: modulation of expression in Cloudman melanoma cells by UVB radiation. J Cell Physiol. 1991;147: 1–6.](http://paperpile.com/b/6W2Kpw/D0P5)

142. [Langendonk JG, Balwani M, Anderson KE, Bonkovsky HL, Anstey AV, Bissell DM, et al. Afamelanotide for Erythropoietic Protoporphyria. N Engl J Med. 2015;373: 48–59.](http://paperpile.com/b/6W2Kpw/H3pO)

143. [He Y, Jones CR, Fujiki N, Xu Y, Guo B, Holder JL Jr, et al. The transcriptional repressor DEC2 regulates sleep length in mammals. Science. 2009;325: 866–870.](http://paperpile.com/b/6W2Kpw/t3Gj)

144. [Hirano A, Hsu P-K, Zhang L, Xing L, McMahon T, Yamazaki M, et al. DEC2 modulates orexin expression and regulates sleep. Proc Natl Acad Sci U S A. 2018;115: 3434–3439.](http://paperpile.com/b/6W2Kpw/3daV)

145. [Pellegrino R, Kavakli IH, Goel N, Cardinale CJ, Dinges DF, Kuna ST, et al. A novel BHLHE41 variant is associated with short sleep and resistance to sleep deprivation in humans. Sleep. 2014;37: 1327–1336.](http://paperpile.com/b/6W2Kpw/gxas)

146. [Shi G, Xing L, Wu D, Bhattacharyya BJ, Jones CR, McMahon T, et al. A Rare Mutation of β1-Adrenergic Receptor Affects Sleep/Wake Behaviors. Neuron. 2019. pp. 1044–1055.e7. doi:](http://paperpile.com/b/6W2Kpw/qnPQ)[10.1016/j.neuron.2019.07.026](http://dx.doi.org/10.1016/j.neuron.2019.07.026)

147. [Xing L, Shi G, Mostovoy Y, Gentry NW, Fan Z, McMahon TB, et al. Mutant neuropeptide S receptor reduces sleep duration with preserved memory consolidation. Sci Transl Med. 2019;11. doi:](http://paperpile.com/b/6W2Kpw/CtCm)[10.1126/scitranslmed.aax2014](http://dx.doi.org/10.1126/scitranslmed.aax2014)
